# Directed evolution of engineered virus-like particles with improved production and transduction efficiencies

**DOI:** 10.1038/s41587-024-02467-x

**Published:** 2024-11-13

**Authors:** Aditya Raguram, Meirui An, Paul Z. Chen, David R. Liu

**Affiliations:** 1 Merkin Institute of Transformative Technologies in Healthcare, Broad Institute of MIT and Harvard, Cambridge, MA USA; 2 Department of Chemistry and Chemical Biology, Harvard University, Cambridge, MA USA; 3 Howard Hughes Medical Institute, Harvard University, Cambridge, MA USA; 4 Koch Institute for Integrative Cancer Research, Massachusetts Institute of Technology, Cambridge, MA USA; 5 Present Address: Whitehead Institute for Biomedical Research, Cambridge, MA USA

**Keywords:** Genetic engineering, Protein delivery

## Abstract

Engineered virus-like particles (eVLPs) are promising vehicles for transient delivery of proteins and RNAs, including gene editing agents. We report a system for the laboratory evolution of eVLPs that enables the discovery of eVLP variants with improved properties. The system uses barcoded guide RNAs loaded within DNA-free eVLP-packaged cargos to uniquely label each eVLP variant in a library, enabling the identification of desired variants following selections for desired properties. We applied this system to mutate and select eVLP capsids with improved eVLP production properties or transduction efficiencies in human cells. By combining beneficial capsid mutations, we developed fifth-generation (v5) eVLPs, which exhibit a 2–4-fold increase in cultured mammalian cell delivery potency compared to previous-best v4 eVLPs. Analyses of v5 eVLPs suggest that these capsid mutations optimize packaging and delivery of desired ribonucleoprotein cargos rather than native viral genomes and substantially alter eVLP capsid structure. These findings suggest the potential of barcoded eVLP evolution to support the development of improved eVLPs.

## Abstract (web summary)

Engineered virus-like particles are improved through directed evolution.

## Main

The ability to safely and efficiently deliver macromolecules into relevant cell populations in culture (in vitro) and in the body (in vivo) is a requirement for many emerging therapeutic modalities. Current gene editing technologies^1,2^, for example, are often constrained by the challenge of delivering gene editing agents into relevant cell types in vitro and in vivo^3–5^. Viral vectors such as adeno-associated virus (AAV) have been used to deliver gene editing agents into several tissues in vivo, including in clinical trials^3,6–12^. However, AAV delivery is limited by cargo size restrictions^13^, the possibility of unwanted DNA cargo integration into the genomes of transduced cells^14,15^ and the prolonged expression of gene editing agents in transduced cells, which increases risks of off-target editing^3,9,16^. Some nonviral delivery methods, including lipid nanoparticles (LNPs), offer reduced off-target editing by transiently delivering editor-encoding mRNA instead of DNA; however, using LNP delivery to achieve therapeutic gene editing in extrahepatic tissues remains challenging^3,17,18^ despite recent encouraging progress^19–22^. Thus, the development of additional delivery strategies is needed to overcome the limitations of these existing methods.

Recently, we and others have explored the use of virus-like particles (VLPs) as vehicles for delivering gene editing agents into cells in vitro or in vivo^3,16,23–35^. VLPs consist of viral scaffolds that package and deliver cargo proteins, ribonucleoproteins (RNPs) or mRNAs instead of cargo-encoding viral genomes. Thus, VLP delivery offers the efficient transduction and tissue tropisms of viral delivery methods with the transient cargo expression and reduced off-target editing of nonviral delivery methods^3,16^, an ideal combination for gene editing applications.

Several VLP-based strategies for delivering gene editing agents into mammalian cells have been previously described^3,16,23–35^. We recently developed engineered VLPs (eVLPs) that enable efficient protein delivery and gene editing in cell culture and in the mouse liver and retina^16^. In eVLPs, desired cargo proteins are fused to retroviral Gag (capsid) proteins, which directs localization of the cargo into viral particles as they form in producer cells. The Gag–cargo linker contains a sequence engineered to be cleaved at a carefully tuned rate by the copackaged retroviral protease following particle formation, which releases the cargo inside the particles and subsequently into the transduced cells. Additionally, the cell-type specificity of eVLPs is determined by the envelope glycoprotein used to pseudotype the particles. By iteratively engineering eVLPs to improve cargo loading, cargo release and component stoichiometry, we developed an optimized fourth-generation (v4) eVLP architecture that was critical for enabling efficient in vivo base editing with minimal off-target editing compared to AAV delivery. We also recently reported prime editor (PE)-eVLPs, which enable transient in vivo delivery of therapeutic PE RNPs with minimal off-target editing and no risk of oncogenic transgene insertion^36^. These favorable characteristics of eVLPs suggest that eVLP delivery has the potential to serve as a useful modality for the in vitro and in vivo delivery of gene editing RNPs or other therapeutic proteins.

Additional improvements to the properties of eVLPs are needed to maximize their potential for research and therapeutic applications. In particular, increasing eVLP packaging efficiency or per-particle transduction efficiency would enable more efficient gene editing with lower eVLP doses. Directed laboratory evolution is a promising approach for improving delivery vehicles and has been used extensively to develop viral delivery vectors with desired properties^3,37–46^. Existing evolution schemes require each viral variant to package a viral genome that encodes that particular variant’s identity, which allows sequencing the viral genomes that survive a selection to identify variants that possess the desired properties. Because eVLPs do not package any viral genetic material, however, applying directed evolution to improve eVLPs requires developing an alternative strategy to encode an eVLP variant’s identity.

To achieve this goal, we developed a directed evolution system in which each eVLP variant packages RNPs loaded with guide RNAs containing a barcode sequence that uniquely identifies that particular eVLP variant. After applying selections for specific properties, desired eVLP variants are identified by sequencing the surviving barcoded guide RNAs. Using this system, we generated a library of eVLP capsid mutants and performed selections to identify capsid mutants that support improved eVLP production from producer cells or improved eVLP transduction of target cells. By combining the beneficial capsid mutations, we developed v5 eVLPs, which exhibit increased RNP packaging, improved cargo release, distinct capsid structural compositions and a 2–4-fold increase in in vitro delivery potency compared to previous-best v4 eVLPs. A key capsid mutation in v5 eVLPs abolishes an interaction that is critical for packaging viral genomes in wild-type viruses but is not required in RNP-packaging eVLPs that lack viral genomes, highlighting the benefits of mutating and explicitly selecting eVLP capsids to package non-native RNP cargos instead of viral genomes. Our results lay a foundation for evolving eVLPs with improved properties.

## Results

### Barcoded guide RNAs identify eVLPs with distinct properties

**Fig. 1:**
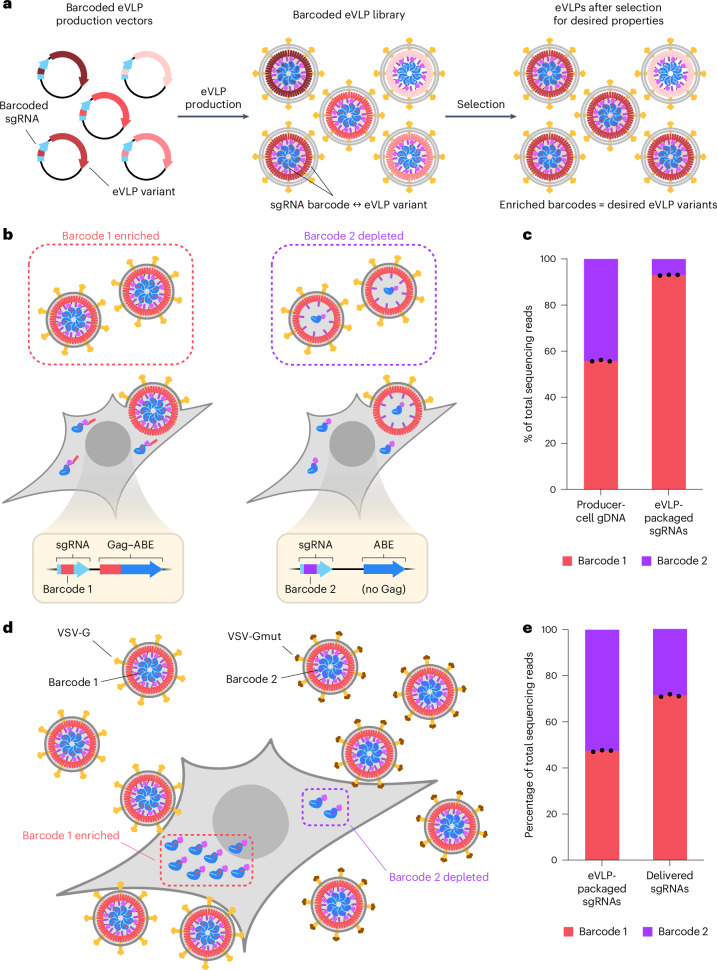
Validation of the barcoded eVLP evolution system. **a**, Overview of the barcoded eVLP evolution system. Each unique eVLP variant is linked to a unique barcoded sgRNA on the same eVLP production vector. eVLP production maintains barcode–variant correspondence and yields a barcoded eVLP library. In the resulting library, each eVLP variant packages RNPs containing barcoded sgRNAs that encode the identity of that particular eVLP variant. Barcodes that are enriched following a selection for desired properties identify eVLP variants that possess the desired properties. **b**, Schematic of the mock production selection experiment with barcode 1 linked to a functional Gag–ABE construct and barcode 2 linked to a nonfunctional ABE only (no Gag) construct. **c**, Frequencies of barcodes 1 or 2 detected in either the producer-cell gDNA or the eVLP-packaged sgRNAs. Bars reflect the mean of *n* = 3 biological replicates and dots represent individual replicate values. **d**, Schematic of the mock transduction selection experiment with barcode 1 linked to wild-type VSV-G envelope protein and barcode 2 linked to an impaired VSV-Gmut envelope protein. **e**, Frequencies of barcodes 1 or 2 detected in either the eVLP-packaged sgRNAs or the delivered sgRNAs. Bars reflect the mean of *n* = 3 biological replicates and dots represent individual replicate values.

All directed evolution systems require a way to identify desired variants following a selection for specific properties^47,48^. To overcome the challenge created by the lack of viral genetic material within eVLPs, we envisioned a strategy to encode the identity of each eVLP variant using barcoded single-guide RNAs (sgRNAs) within eVLP-packaged RNP cargos (Fig. 1a). In this scheme, each eVLP production vector expresses both an eVLP variant and a barcoded sgRNA that uniquely identifies that eVLP variant (Fig. 1a). These barcoded eVLP production vectors are introduced into producer cells under conditions that maximize the likelihood that each producer cell receives only a single barcoded vector and, therefore, produces only a single eVLP variant–barcoded sgRNA combination. This strategy generates barcoded eVLP libraries in which each unique eVLP variant packages sgRNAs containing a unique corresponding barcode (Fig. 1a).

After subjecting a barcoded eVLP library to a selection for a desired property, eVLP variants surviving selection are identified by sequencing their sgRNAs and determining which barcodes are enriched in the postselection population compared to the input population (Fig. 1a). This scheme for evolving barcoded eVLPs in principle can be used to evolve different eVLP components—including capsid, envelope, cargo and other structural proteins—by placing the evolving component on the same vector as the barcoded sgRNA when constructing the library of eVLP production vectors (Fig. 1a). Additionally, this scheme is compatible with a wide range of selections for desired properties, including improved particle production, particle stability or transduction of a particular cell type in vitro or in vivo.

**Extended Data Fig. 1:**
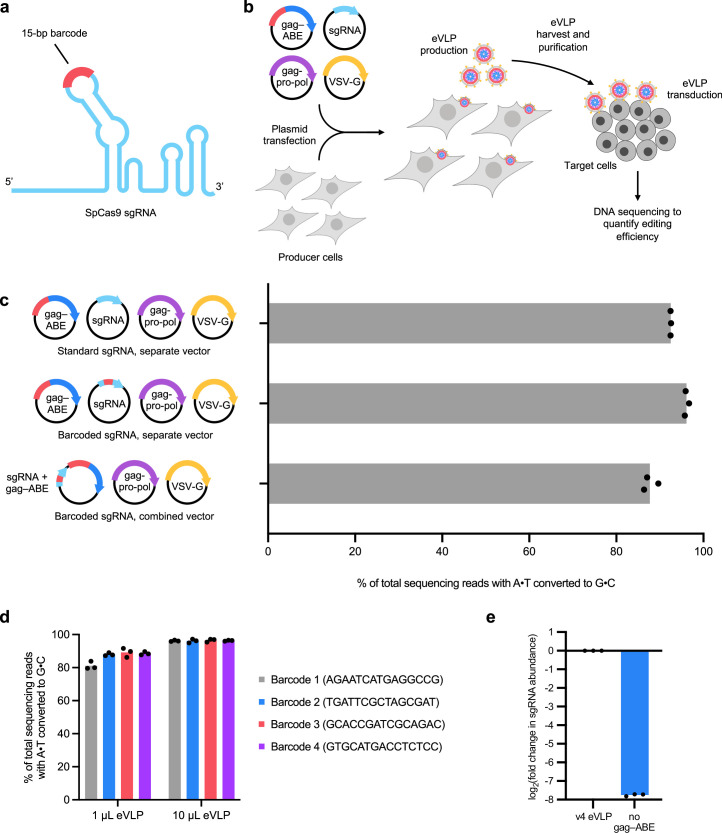
Barcoded sgRNAs are compatible with eVLPs. **a**, Schematic of the 15-bp barcode sequence (in red) inserted into the tetraloop of the SpCas9 sgRNA scaffold. **b**, Overview of the standard eVLP production workflow. **c**, Adenine base editing efficiencies at position A_7_ of the *BCL11A* enhancer site in HEK293T cells transduced by eVLPs produced using a four-plasmid transfection or three-plasmid (combined sgRNA+gag–ABE vector) transfection and either standard or barcoded sgRNAs as shown. **d**, Adenine base editing efficiencies at position A_7_ of the *BCL11A* enhancer site in HEK293T cells transduced by eVLPs produced using one of four arbitrarily selected barcoded sgRNAs. **e**, Fold change in eVLP-packaged sgRNA abundance measured by RT-qPCR using sgRNA-specific primers, normalized relative to sgRNA abundance in v4 eVLPs. **c–e**, Bars reflect the mean of n = 3 biological replicates, and dots represent individual replicate values.

We first validated that barcoded sgRNAs are compatible with functional eVLP production. We inserted a 15-bp barcode sequence into the tetraloop of the sgRNA scaffold (Extended Data Fig. 1a), as previous studies showed that this location and length of insertion does not disrupt sgRNA function^49,50^. For all validation experiments, we used our previously developed v4 base editor (BE)-eVLPs^16^ that package a highly active adenine base editor (ABE8e) RNP cargo^51^. Standard v4 BE-eVLPs are produced by cotransfecting four expression plasmids into producer cells (Extended Data Fig. 1b), encoding the expression of (1) the Gag–ABE fusion; (2) the sgRNA that directs on-target base editing in the transduced cells; (3) the Moloney murine leukemia virus (MMLV) Gag–Pro–Pol polyprotein, which contains the required viral protease and other structural components; and (4) the vesicular stomatitis virus G (VSV-G) envelope protein.

We produced v4 eVLPs containing canonical or tetraloop-barcoded sgRNAs with four arbitrarily chosen barcodes and compared their potencies by measuring base editing efficiencies at the *BCL11A* enhancer locus in eVLP-transduced HEK293T cells. We observed that barcoded eVLPs exhibited comparable potency to standard eVLPs and that eVLPs produced with distinct barcoded sgRNAs exhibited comparable potencies (Extended Data Fig. 1c,d). Because the evolution scheme requires that the barcoded sgRNA and evolving eVLP component are expressed from the same vector, we also confirmed that a single vector containing both an sgRNA expression cassette and a Gag–ABE fusion could support efficient eVLP production and cargo delivery (Extended Data Fig. 1c). Reverse transcription quantitative PCR (RT–qPCR) analysis confirmed that eVLPs lacking Gag–ABE package 216-fold fewer sgRNA molecules compared to canonical v4 eVLPs, suggesting that sgRNA packaging in the absence of Gag–ABE is negligible and, therefore, not likely to influence selection outcomes (Extended Data Fig. 1e). These results together indicate that barcoded BE-eVLPs can be produced in a manner that preserves standard BE-eVLP properties.

Lastly, we validated that barcoded eVLPs can be used to distinguish between eVLP variants with different functional properties. To do so, we performed two mock selections: a selection for cargo-loaded eVLP production and a selection for eVLP transduction of HEK293T cells. We performed the mock eVLP production selection using two different BE-eVLP cargo constructs: (1) a standard Gag–ABE cargo construct used in v4 eVLPs and (2) a nonfunctional cargo construct containing an ABE but no Gag fusion, which almost completely abolishes ABE cargo loading into eVLPs^16^. We paired each of these two cargo constructs with a unique barcoded sgRNA and used lentiviral integration to generate producer cells expressing either barcode 1 (corresponding to Gag–ABE) or barcode 2 (corresponding to ABE only) (Fig. 1b). We then initiated eVLP production from a 1:1 mixture of these producer cells. Because only the barcode 1 (Gag–ABE) producer cells and not barcode 2 (ABE only) producer cells can produce functional eVLPs containing substantial amounts of ABE RNP cargo, we anticipated that barcode 1 would be enriched in eVLPs, while barcode 2 would be depleted (Fig. 1b). Indeed, we observed that barcode 1 was strongly enriched 13-fold (93% of sequencing reads) compared to barcode 2 (7% of sequencing reads) in eVLP-packaged sgRNAs even though barcodes 1 and 2 were equally represented in the original producer cell mixture (Fig. 1c).

Next, we performed a mock eVLP transduction selection using two different eVLP envelope constructs: (1) a standard VSV-G envelope construct that enables transduction of most cell types, and (2) an impaired VSV-Gmut envelope construct that reduces but does not completely eliminate the ability of viral particles to engage target cell surface receptors and deliver their packaged cargo in the absence of additional targeting ligands^34,52,53^. We produced VSV-G-pseudotyped eVLPs packaging barcode 1 and VSV-Gmut-pseudotyped eVLPs packaging barcode 2 and then transduced HEK293T cells with a 1:1 mixture of these barcoded eVLPs (Fig. 1d). Because VSV-G-pseudotyped eVLPs should more efficiently transduce cells and deliver RNP cargo compared to VSV-Gmut-pseudotyped eVLPs, we anticipated that barcode 1 would be enriched in sgRNAs retrieved from eVLP-transduced cells compared to sgRNAs packaged in the input eVLP mixture while barcode 2 would be depleted (Fig. 1d). Indeed, we observed that barcode 1 was enriched 2.4-fold (71% of sequencing reads) compared to barcode 2 (29% of sequencing reads) in sgRNAs retrieved from eVLP-transduced cells even though barcodes 1 and 2 were equally represented in the original eVLP mixture (Fig. 1e).

These results of the mock eVLP production selection and mock eVLP transduction selection demonstrate that barcoded sgRNAs can be used to label different eVLP variants and that barcodes that are enriched following a selection identify variants with increased fitness. Collectively, these findings validate key aspects of the barcoded eVLP evolution system and establish a framework for using barcoded sgRNAs to identify eVLP variants with desired properties.

### Mutating and selecting a barcoded eVLP capsid library

Next, we applied the barcoded eVLP evolution system to mutate and select eVLP capsids with improved properties. The capsid proteins that are used in v4 eVLPs are identical to the capsid proteins used in wild-type MMLV, which have evolved in nature to be optimal for packaging viral genomes^54,55^. Therefore, wild-type MMLV capsids are likely not optimal for packaging large, non-native protein cargos such as ABEs in eVLPs. We hypothesized that remodeling the internal eVLP capsid surface to optimize ABE RNP cargo packaging instead of viral genome packaging could substantially improve eVLP properties, including potency per particle, number of cargo molecules packaged per particle, overall particle yield or titer and particle stability.

**Extended Data Fig. 2:**
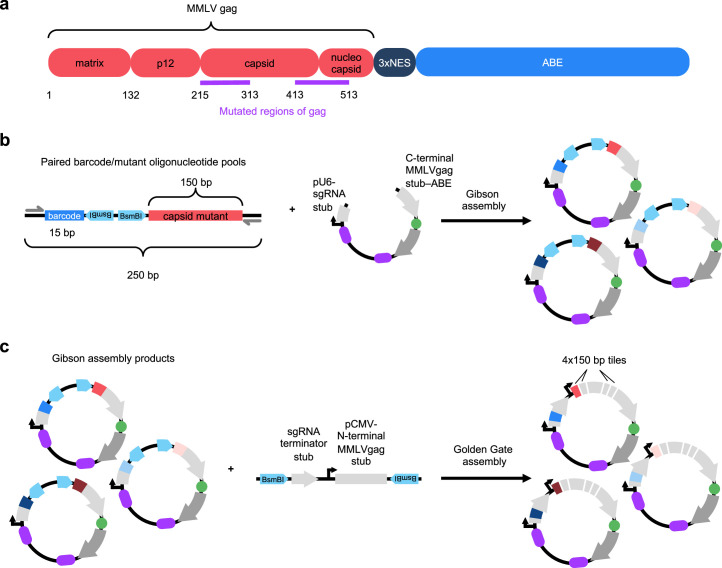
Barcoded eVLP capsid library construction. **a**, Schematic of the region of gag that was mutated in the eVLP capsid library. The mutated regions, underlined in purple, spanned 198 total residues of the capsid and nucleocapsid domains of gag. Each residue was mutated to each of the 19 possible non-wild-type residues at each position, resulting in a total library size of 3,762 single-residue mutants plus the canonical v4 eVLP. **b**, Overview of the first step of the barcoded capsid library cloning procedure. Each capsid mutant was synthesized with a 15-bp barcode on the same short oligonucleotide and cloned into an appropriate vector backbone by Gibson assembly. **c**, Overview of the second step of the barcoded capsid library cloning procedure. The Gibson assembly products from the first cloning step were subjected to a Golden Gate assembly reaction to install the intervening promoters and gene sequences in between the barcoded sgRNA and capsid mutant sequences. These two cloning steps were repeated independently four times to generate four sub-libraries, with each sub-library containing all capsid mutants within a 150 bp region of gag.

To mutate and select eVLP capsids to become more optimal for packaging ABE RNP cargo, we first designed and constructed a barcoded eVLP capsid library. This library contained 3,762 single-residue mutants of the MMLV Gag protein capsid (amino acids 215–313 and 413–479) and nucleocapsid (amino acids 480–513) domains in the Gag–ABE cargo construct (Extended Data Fig. 2a). We implemented a library construction strategy that preserved the association between barcodes and mutants to enable decoding of selection outcomes (Extended Data Fig. 2b,c). Barcodes were chosen such that no two barcode sequences were within four mismatches of each other to minimize the likelihood of incorrect barcode classification because of sequencing errors during barcode retrieval or mutations during eVLP production.

**Fig. 2:**
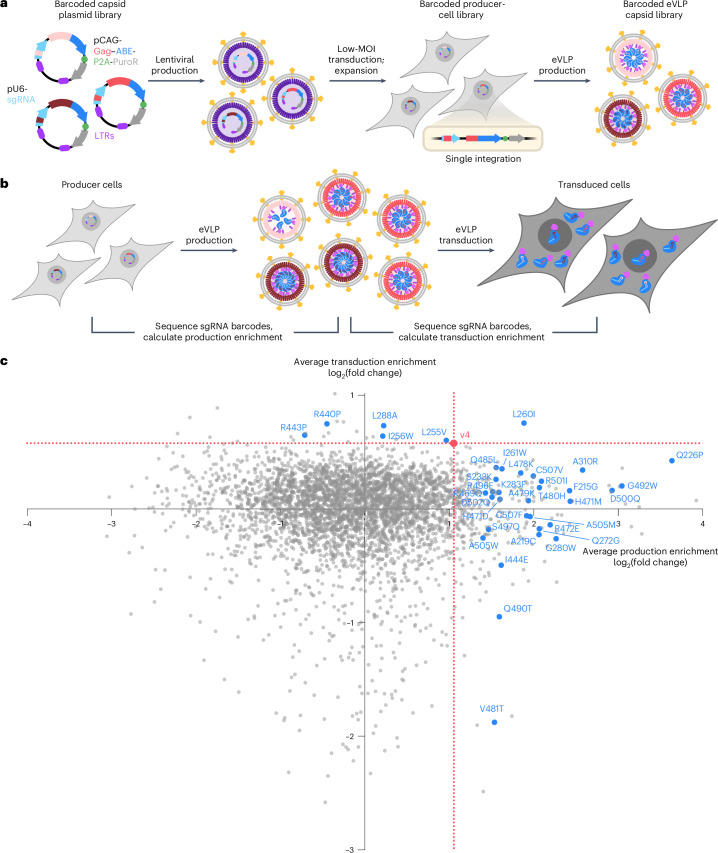
Barcoded eVLP capsid selections. **a**, Schematic of the barcoded eVLP capsid library generation. Each unique capsid mutant was linked to a unique barcoded sgRNA on the same plasmid vector. These barcoded vectors were used to produce lentivirus, which was then used to generate a barcoded producer cell library in which each producer cell contained a single integration of a barcoded sgRNA and capsid mutant expression cassette. Following expansion of transduced cells, the barcoded producer cell library was transfected with the other plasmids necessary for eVLP production to generate a barcoded eVLP capsid library. **b**, Overview of selections for improved eVLP production and improved eVLP transduction. Barcodes enriched in eVLP-packaged sgRNAs relative to producer-cell gDNA identify capsid mutants that support improved eVLP production. Barcodes enriched in eVLP-transduced cells relative to eVLP-packaged sgRNAs identify capsid mutants that support improved eVLP transduction. **c**, Average barcode enrichment values for each capsid mutant in the production selection and transduction selection. Each capsid mutant is shown as a single dot whose *x* coordinate reflects the average production enrichment of the capsid mutant and *y* coordinate reflects the average transduction enrichment of the capsid mutant. The canonical capsid used in v4 eVLPs is shown as a red dot and the corresponding enrichment values associated with this dot are shown as dotted red lines. Capsid mutants selected for further characterization are shown as blue dots. Production and transduction enrichment values were calculated as the average of *n* = 2 replicates. Further details are provided in Extended Data Figs. 4 and 5 and Supplementary Table 3.

**Extended Data Fig. 3:**
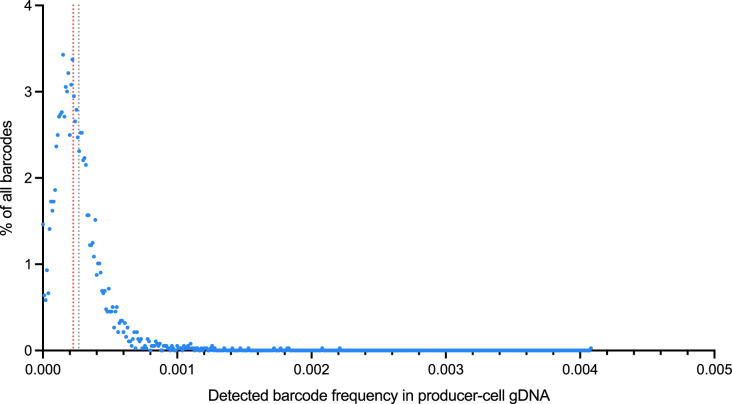
Barcode frequency distribution in producer-cell gDNA. Distribution of barcode frequencies detected in producer-cell gDNA. The theoretical median of this frequency distribution, assuming perfectly equal representation of all library members, is depicted by a dotted grey line at $x=\frac{1}{3763}\approx 0.000266$. The observed median (0.000225) is depicted by a dotted red line.

We used this barcoded plasmid library to generate a library of barcoded eVLP producer cells (Fig. 2a). Lentiviral transduction of producer cells at a low multiplicity of infection (MOI) followed by expansion of the transduced cells maximized the fraction of producer cells that each received a single barcode–capsid variant pair (Supplementary Table 5). High-throughput sequencing analysis of genomic DNA (gDNA) isolated from the expanded producer cell library revealed that 99% of all barcode sequences were detected (Extended Data Fig. 3). These results demonstrate the successful generation of barcoded eVLP plasmid and producer cell libraries, laying the foundation for eVLP evolution campaigns.

### Selections reveal how capsid mutants affect eVLP properties

**Extended Data Fig. 4:**
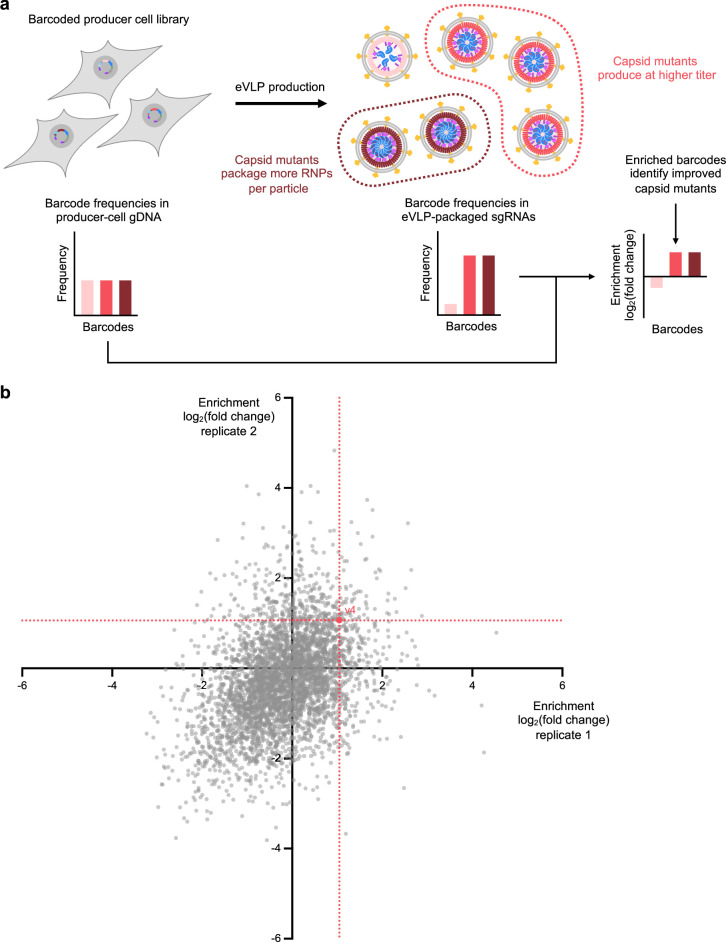
Selecting eVLP capsid mutants with improved production. **a**, Schematic of the production selection. The production enrichment value for each barcode was calculated by dividing the frequency of that barcode in the eVLP-packaged sgRNAs by the frequency of that barcode in the producer-cell gDNA. Enriched barcodes identify capsid mutants that support improved production. **b**, Production enrichment values for all assessed capsid mutants. Each capsid mutant is shown as a single dot whose x-coordinate reflects that mutant’s enrichment in production selection replicate 1 and y-coordinate reflects that mutant’s enrichment in production selection replicate 2. The canonical capsid used in v4 eVLPs is shown as a red dot, and the corresponding enrichment values associated with this dot are shown as dotted red lines.

We subjected the barcoded eVLP capsid library to two separate selections (Fig. 2b): (1) a selection for improved eVLP production from producer cells, and (2) a selection for improved eVLP transduction of HEK293T cells. To perform a selection for improved eVLP production, we initiated eVLP production from the barcoded producer cell library (Methods). We purified the resulting library of barcoded eVLP capsid variants, isolated the eVLP-packaged sgRNAs and sequenced the barcodes that were present after this production selection. For each barcode sequence in the library, we calculated the eVLP production enrichment by comparing the frequency of that barcode in eVLP-packaged sgRNAs to the frequency of that barcode in the producer-cell gDNA. In this production selection, barcodes that display greater enrichment than the canonical eVLP capsid barcode identify candidate capsid mutants that support improved production compared to the canonical capsid (Extended Data Fig. 4a). Enriched barcodes, for example, might indicate that those capsid mutants package more RNP cargo molecules per particle than the canonical capsid or are produced at a higher titer, either of which could explain why those particular sgRNAs were more abundant in the produced eVLPs relative to producer-cell gDNA.

Approximately 8% of all capsid mutants in the library exhibited an average production enrichment higher than that of the canonical eVLP capsid (Fig. 2c and Extended Data Fig. 4b). Because the complete MMLV capsid consists of a complex assembly of thousands of capsid subunits^54^, it is likely that the majority of capsid mutations disrupt the carefully orchestrated process of capsid assembly, explaining the rarity of mutants enriched beyond that of the canonical eVLP capsid in the eVLP production selection. The enrichment of a subpopulation of capsid mutants in the production selection beyond canonical eVLPs (Fig. 2c and Extended Data Fig. 4b), however, supported our hypothesis that the wild-type MMLV capsid is not optimal for RNP cargo packaging and that eVLP capsids can be mutated and selected in the laboratory to improve this property.

**Extended Data Fig. 5:**
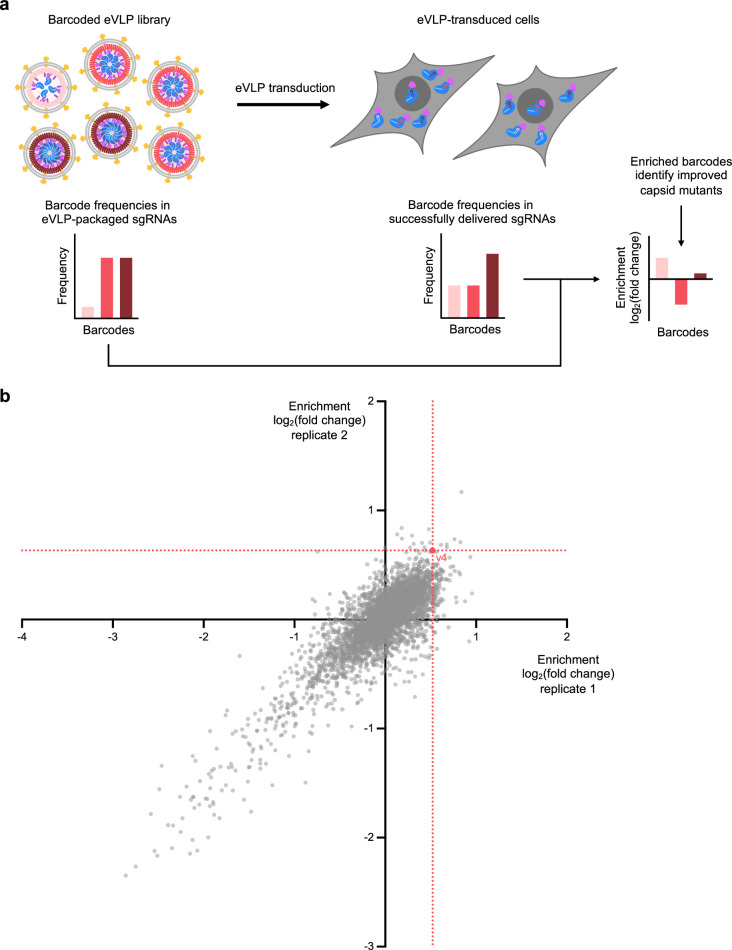
Selecting eVLP capsid mutants with improved transduction. **a**, Schematic of the transduction selection. The transduction enrichment value for each barcode was calculated by dividing the frequency of that barcode in the successfully delivered sgRNAs by the frequency of that barcode in the eVLP-packaged sgRNAs. Enriched barcodes identify capsid mutants that support improved transduction. **b**, Transduction enrichment values for all assessed capsid mutants. Each capsid mutant is shown as a single dot whose x-coordinate reflects that mutant’s enrichment in transduction selection replicate 1 and y-coordinate reflects that mutant’s enrichment in transduction selection replicate 2. The canonical capsid used in v4 eVLPs is shown as a red dot, and the corresponding enrichment values associated with this dot are shown as dotted red lines.

In addition to improving eVLP production, the eVLP evolution system can be used to improve transduction of eVLPs into target cells (Fig. 2b). We incubated HEK293T cells with the purified barcoded eVLP capsid library and isolated sgRNAs that were successfully transduced into target cells after 6 h. For each barcode sequence in the library, we calculated the eVLP transduction enrichment by comparing the frequency of that barcode in the transduced HEK293T cells to the frequency of that barcode in the eVLP-packaged sgRNAs before incubation with HEK293T cells. Barcodes that are enriched to a higher degree than the canonical v4 eVLP barcode identify capsid mutants that support improved transduction relative to the v4 eVLP capsid (Extended Data Fig. 5a). Enriched barcodes, for example, might reflect capsid mutants that transduce target cells more efficiently because they are more stable or enter target cells more efficiently. Notably, we observed that only 0.7% of all capsid mutants in the library exhibited an average transduction enrichment greater than that of the canonical v4 eVLP capsid (Fig. 2c and Extended Data Fig. 5b). These findings support a model in which capsid mutants are more likely to improve eVLP production or RNP cargo packaging but rarely improve particle stability, cell entry or other characteristics that influence transduction.

By integrating the results from both the production and the transduction selections, we generated a landscape that reveals how each capsid mutant influences these two properties of eVLPs (Fig. 2c). The vast majority of capsid mutants exhibited worse production and transduction efficiencies compared to the canonical v4 eVLP capsid. While a handful of mutants showed selection enrichments that suggest improvements in either production or transduction, virtually no mutants exhibited improvements in both properties, suggesting that eVLP production and transduction efficiencies are dictated by distinct and potentially competing mechanisms. Certain clusters of mutations consistently impacted eVLP production and transduction. For example, R440P or R443P improved transduction but negatively impacted production (Fig. 2c). Conversely, L478K, A479K or T480H improved production but modestly impaired transduction (Fig. 2c). These observations suggest that remodeling the internal charged surfaces of the eVLP capsid is a potential strategy for optimizing eVLP capsids to better package and deliver RNP cargo. Together, the results of the eVLP capsid selections demonstrate the utility of the barcoded eVLP system, reveal new insights into how different capsid mutations influence eVLP properties and nominate potentially improved capsid mutants that warrant further characterization.

### Combinations of capsid mutations improve eVLP potency

On the basis of the results of the production and transduction selections, we identified a set of 36 capsid mutants for further characterization (blue dots in Fig. 2c). We chose these mutants on the basis of their positive enrichments in both replicates of the production or transduction selections, prioritizing mutants that improved one property without substantially impairing the other property (Fig. 2c). To perform a high-throughput assessment of the potency of multiple different variants simultaneously, we produced different eVLP variants through transient transfection of eVLP plasmids into producer cells in different wells of 96-well plates, transduced HEK293T cells with the same volume of each eVLP variant at a subsaturating dose (Methods) and determined each the potency of each variant by measuring adenine base editing efficiencies at the sgRNA-specified target *BCL11A* enhancer locus in the transduced cells. These eVLP production conditions impose an eVLP stoichiometry of 25:75 Gag–ABE:Gag–Pro–Pol, a stoichiometry that we previously determined to be optimal for eVLP potency^16^. This 25:75 stoichiometry likely differs from the stoichiometry imposed during the barcoded library selection conditions because eVLP production from singly integrated Gag–ABE producer cell lines likely results in a low Gag–ABE:Gag–Pro–Pol ratio. While we anticipated that this stoichiometry difference might lead to differences between an individual mutant’s performance in selections versus in potency assays, we chose to compare all mutants to canonical v4 eVLPs using optimal transfection-based production conditions to assess whether any of these mutants could outperform v4 eVLPs in this most relevant setting.

**Extended Data Fig. 6:**
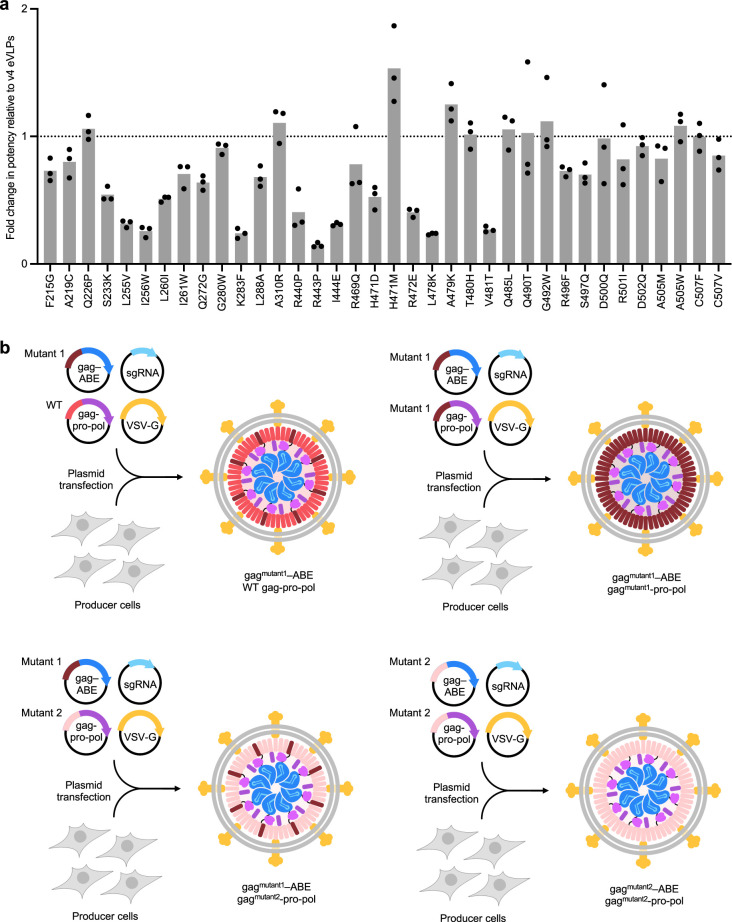
Incorporating capsid mutations into gag–ABE or gag-pro-pol. **a**, Fold change in eVLP potency relative to v4 eVLPs of each capsid mutation incorporated individually into the gag–ABE construct and paired with the wild-type MMLV gag-pro-pol. Bars reflect the mean of n = 3 biological replicates, and dots represent individual replicate values. **b**, Schematic of incorporating different combinations of capsid mutations into either the gag–ABE or gag-pro-pol or both.

We began by introducing each of the 36 capsid mutants into the v4 Gag–ABE construct and used canonical versions of the other components of the v4 eVLP architecture (wild-type MMLV Gag–Pro–Pol, VSV-G and standard sgRNA) to produce the evolved eVLP variants. In this context, we observed that most of the mutations did not improve eVLP potency compared to v4 eVLPs (Extended Data Fig. 6a and Supplementary Fig. 1a), indicating that incorporating the capsid mutations into the Gag–ABE construct alone was not sufficient to improve potency.

**Fig. 3:**
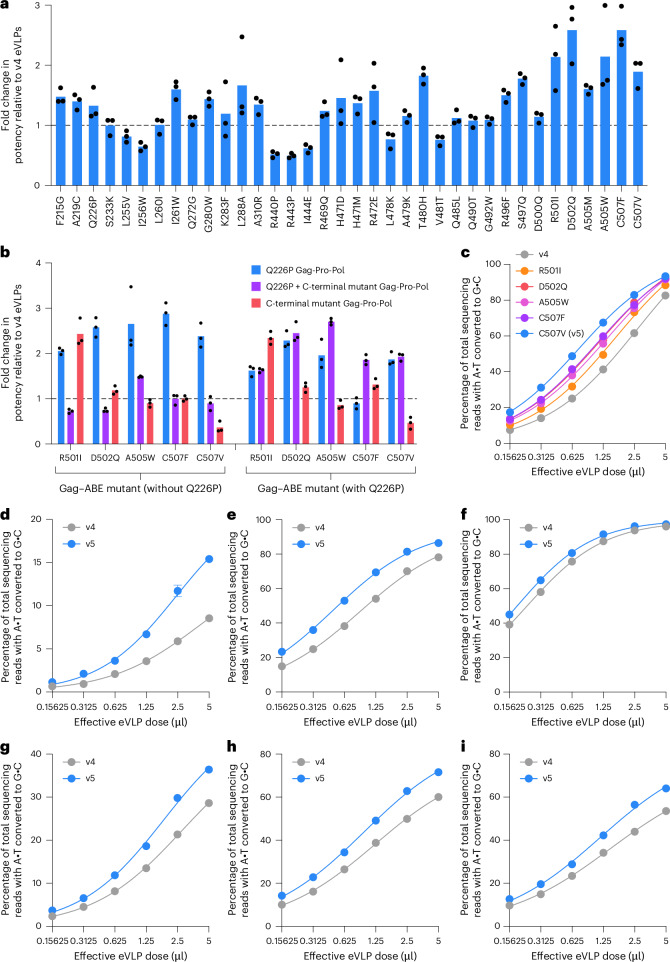
Combinations of capsid mutations improve eVLP potency. **a**, Fold change in eVLP potency compared to v4 eVLPs of each capsid mutant incorporated individually into the Gag–ABE construct and paired with Gag^Q226P^–Pro–Pol. **b**, Fold change in eVLP potency relative to v4 eVLPs of each C-terminal capsid mutant with or without the Q226P mutant incorporated into either the Gag–ABE only, Gag–Pro–Pol only or both Gag–ABE and Gag–Pro–Pol. In **a**,**b**, bars reflect the mean of *n* = 3 biological replicates and dots represent individual replicate values. **c**, Comparison of v4 eVLPs and Gag–ABE mutants paired with the Gag^Q226P^–Pro–Pol across a range of eVLP doses. Adenine base editing efficiencies at position A_7_ of the *BCL11A* enhancer site in HEK293T cells are shown. eVLPs were produced at a concentration of approximately 5 × 10^8^ eVLPs per microlitre. **d**–**i**, Comparison of v4 and v5 eVLPs in mouse N2A cells across a range of eVLP doses. Adenine base editing efficiencies are shown at position A_8_ of the *Angptl3* exon 7 splice acceptor site (**d**), position A_4_ of the *Rosa26* site (**e**), position A_9_ of the *Dnmt1* site (**f**), position A_6_ of the *Pcsk9* exon 4 splice acceptor site (**g**), position A_4_ of the *Pcsk9* exon 6 splice donor site (**h**) and position A_8_ of the *Pcsk9* exon 8 splice acceptor site (**i**). In **c**–**i**, dots and error bars represent the mean ± s.e.m. of *n* = 3 biological replicates. Data were fit to four-parameter logistic curves using nonlinear regression.

**Extended Data Fig. 7:**
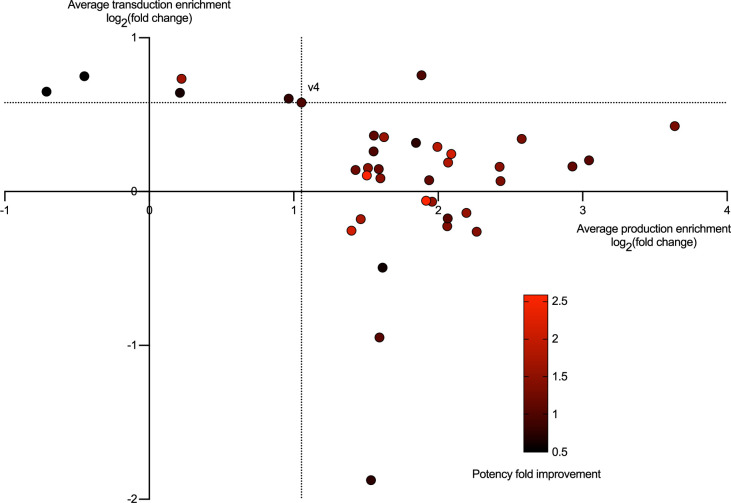
Characterizing the production enrichment, transduction enrichment, and fold improvement in potency of eVLPs containing capsid mutations. Each capsid mutant is shown as a single dot whose x-coordinate reflects that capsid mutant’s average production enrichment and y-coordinate reflects that capsid mutant’s average transduction enrichment (data from Fig. 2c). The color of each dot reflects the fold improvement in potency relative to v4 eVLPs (data from Fig. 3a).

Because the processed Gag protein expressed in the Gag–Pro–Pol construct, along with the processed Gag protein expressed in the Gag–ABE construct, are both important contributors to the overall eVLP capsid, we also incorporated the capsid mutations into the Gag–Pro–Pol construct used for eVLP production (Extended Data Fig. 6b). We first incorporated the Q226P mutation into the Gag–Pro–Pol construct (hereafter referred to as Gag^Q226P^–Pro–Pol), because the Q226P mutation was the most strongly enriched mutation from the production selection that only modestly impaired transduction (Fig. 2c). Next, we assessed the potency of the same 36 capsid mutants in the Gag–ABE construct but now using the Gag^Q226P^–Pro–Pol instead of the wild-type MMLV Gag–Pro–Pol. In this context, many of the tested capsid mutants exhibited 2–3-fold increases in BE delivery potency compared to v4 eVLPs (Fig. 3a and Supplementary Fig. 1b). In general, we observed that mutants that modestly improved production exhibited greater improvements in potency compared to mutants that substantially improved production (Extended Data Fig. 7), consistent with a model in which excessive improvements in eVLP production or cargo packaging might be detrimental to overall eVLP potency.

In light of the discovery that different Gag–ABE and Gag–Pro–Pol capsid mutants can synergize, we systematically evaluated the effects of incorporating different combinations of mutations into the Gag–ABE or Gag–Pro–Pol constructs. We selected five Gag–ABE mutants that exhibited the highest potency when paired with the Gag^Q226P^–Pro–Pol: R501I, D502Q, A505W, C507F and C507V (Fig. 3a). We tested all possible combinations of each C-terminal mutant and Q226P mutant incorporated into the Gag–ABE only, Gag–Pro–Pol only or both Gag–ABE and Gag–Pro–Pol (Fig. 3b). Notably, four of the five C-terminal Gag–ABE mutants still performed best when paired with the Gag^Q226P^–Pro–Pol instead of a matched Gag–Pro–Pol containing that same C-terminal mutant (Fig. 3b). These findings reveal the complex interplay between different capsid mutations and underscore the importance of assessing these mutations in several possible eVLP configurations.

We next evaluated the potency of these five eVLP variants (R501I, D502Q, A505W, C507F or C507V Gag–ABE mutants paired with Gag^Q226P^–Pro–Pol) compared to v4 eVLPs across a range of doses in HEK293T cells (Fig. 3c). As expected, all eVLP variants exhibited improved base editing efficiencies at all doses tested compared to v4 eVLPs (Fig. 3c). In particular, the Gag^C507V^–ABE + Gag^Q226P^–Pro–Pol combination exhibited an average overall 3.7-fold improvement in potency (half-maximal effective concentration, EC_50_) (Fig. 3c). This substantial improvement in potency is comparable to what we observed between v2 and v1 eVLPs or v3 and v2 eVLPs in our previous study^16^. Therefore, we designated the Gag^C507V^–ABE + Gag^Q226P^–Pro–Pol combination as v5 BE-eVLPs (Fig. 3c).

**Extended Data Fig. 8:**
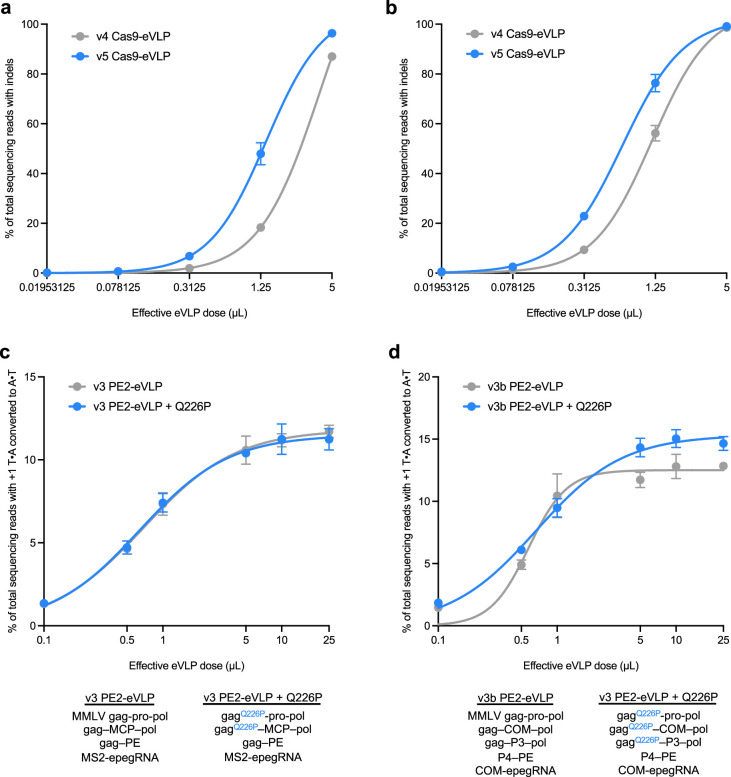
Incorporating capsid mutations into Cas9- or PE-eVLPs. **a–b**, Comparison of v4 Cas9-eVLPs and v5 Cas9-eVLPs (gag^Q226P^-pro-pol/gag^C507V^–Cas9) across a range of eVLP doses. Indel frequencies at *BCL11A* enhancer site (**a**) or the *EMX1* site (**b**) in HEK293T cells are shown. **c**, Comparison of v3 PE2-eVLPs and v3 PE2-eVLPs containing the Q226P mutation incorporated into gag-pro-pol components across a range of eVLP doses. +1 T•A-to-A•T editing efficiencies (without indels) at the *HEK3* site in HEK293T cells are shown. **d**, Comparison of v3b PE2-eVLPs and v3b PE2-eVLPs containing the Q226P mutation incorporated into gag-pro-pol components across a range of eVLP doses. +1 T•A-to-A•T editing efficiencies (without indels) at the *HEK3* site in HEK293T cells are shown. **a–d**, Dots and error bars represent mean ± s.e.m. of n = 3 biological replicates. Data were fit to four-parameter logistic curves using nonlinear regression.

We also investigated whether incorporating mutations identified in the BE-eVLP selections might also improve the delivery potency of eVLPs that package other gene editing cargos, such as Cas9 nuclease or PEs. We observed that Cas9-eVLPs containing Gag^C507V^–Cas9 + Gag^Q226P^–Pro–Pol exhibited an average twofold improvement in potency (EC_50_ value) compared to v4 Cas9-eVLPs at both the *BCL11A* enhancer site and the *EMX1* site in HEK293T cells (Extended Data Fig. 8a,b). Therefore, we designated the Gag^C507V^–Cas9 + Gag^Q226P^–Pro–Pol combination as v5 Cas9-eVLPs. We also observed that v3 or v3b PE2-eVLPs^36^ containing the Q226P mutation incorporated into the relevant Gag–Pro–Pol constructs exhibited comparable potency to canonical v3 or v3b PE2-eVLPs (Extended Data Fig. 8c,d). Together, these results suggest that mutations identified in the BE-eVLP selections can similarly improve the delivery potency of Cas9-eVLPs, which are highly similar in architecture to BE-eVLPs, but not PE-eVLPs, which contain multiple additional structural components and RNA-binding proteins specific to PE cargos that might behave differently when combined with the evolved capsid mutants. These findings also raise the possibility that PE-eVLPs or other eVLPs with distinct architectures might benefit from cargo-specific or architecture-specific production and transduction selections that are analogous to those we performed using BE-eVLPs in this study.

Lastly, we evaluated the potency of v5 BE-eVLPs compared to v4 BE-eVLPs across a range of doses and target genomic loci in mouse Neuro-2a (N2A) cells (Fig. 3d–i). In all tested cases, v5 BE-eVLPs exhibited improved potency compared to v4 BE-eVLPs. In general, the base editing efficiency at a given dose of v4 BE-eVLPs can be achieved using a 2–4-fold lower dose of v5 BE-eVLPs (Fig. 3c–i). We anticipate that v5 eVLPs will be especially useful for eVLP delivery applications that are limited by the maximum administrable dose. Collectively, these results demonstrate that the barcoded eVLP evolution system successfully generated improved eVLP capsid mutants that enabled the discovery of v5 BE-eVLPs with improved delivery potency compared to previous-best v4 BE-eVLPs.

### v5 eVLPs improve base editing potency in primary human HSPCs

To further investigate the potential utility of v5 eVLPs, we compared the potencies of v4 and v5 BE-eVLPs in primary human hematopoietic stem and progenitor cells (HSPCs). Ex vivo gene editing of autologous HSPCs followed by transplantation has proven to be powerful approach for treating blood disorders, including sickle cell disease and β-thalassemia^56–61^. Nuclease-mediated disruption of the *BCL11A* erythroid-specific enhancer in HSPCs leads to the induction of fetal hemoglobin expression in erythrocytes, which is sufficient to rescue disease phenotypes associated with these blood disorders and is the first US Food and Drug Administration-approved gene editing drug^56,61,62^. Therapeutic induction of fetal hemoglobin has also been achieved using ex vivo cytosine or adenine base editing to install precise single-base conversions within the *BCL11A* erythroid-specific enhancer or the fetal hemoglobin (*HBG*) promoter in HSPCs^57,63,64^. Because base editing avoids negative consequences associated with nuclease-generated DNA double-strand breaks and uncontrolled mixtures of indel products^57^, base editing-mediated disruption of the *BCL11A* erythroid-specific enhancer in HSPCs could potentially offer advantages over nuclease-mediated disruption.

**Fig. 4:**
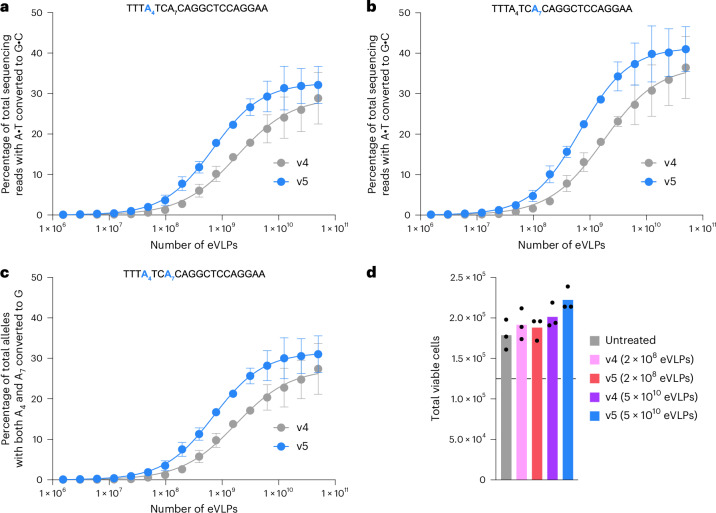
v5 eVLPs improve base editing potency in primary human HSPCs. **a**–**c**, Comparison of v4 and v5 BE-eVLPs in primary human HSPCs across a range of eVLP doses: adenine base editing efficiencies at position A_4_ of the *BCL11A* enhancer site (**a**), position A_7_ of the *BCL11A* enhancer site (**b**) and both positions A_4_ and A_7_ of the *BCL11A* enhancer site (**c**). In **a**–**c**, dots and error bars represent the mean ± s.e.m. of *n* = 3 biological replicates. Data were fit to four-parameter logistic curves using nonlinear regression. The number of eVLPs was quantified using anti-MLV p30 ELISAs (Methods). Plots showing individual replicate values are provided in Extended Data Fig. 9. **d**, Total viable cell counts measured 48 h after eVLP transduction. The dotted line denotes the initial number of cells measured in each condition (1.25 × 10^5^ cells) immediately before eVLP transduction. Bars reflect the mean of *n* = 3 biological replicates and dots represent individual replicate values.

**Extended Data Fig. 9:**
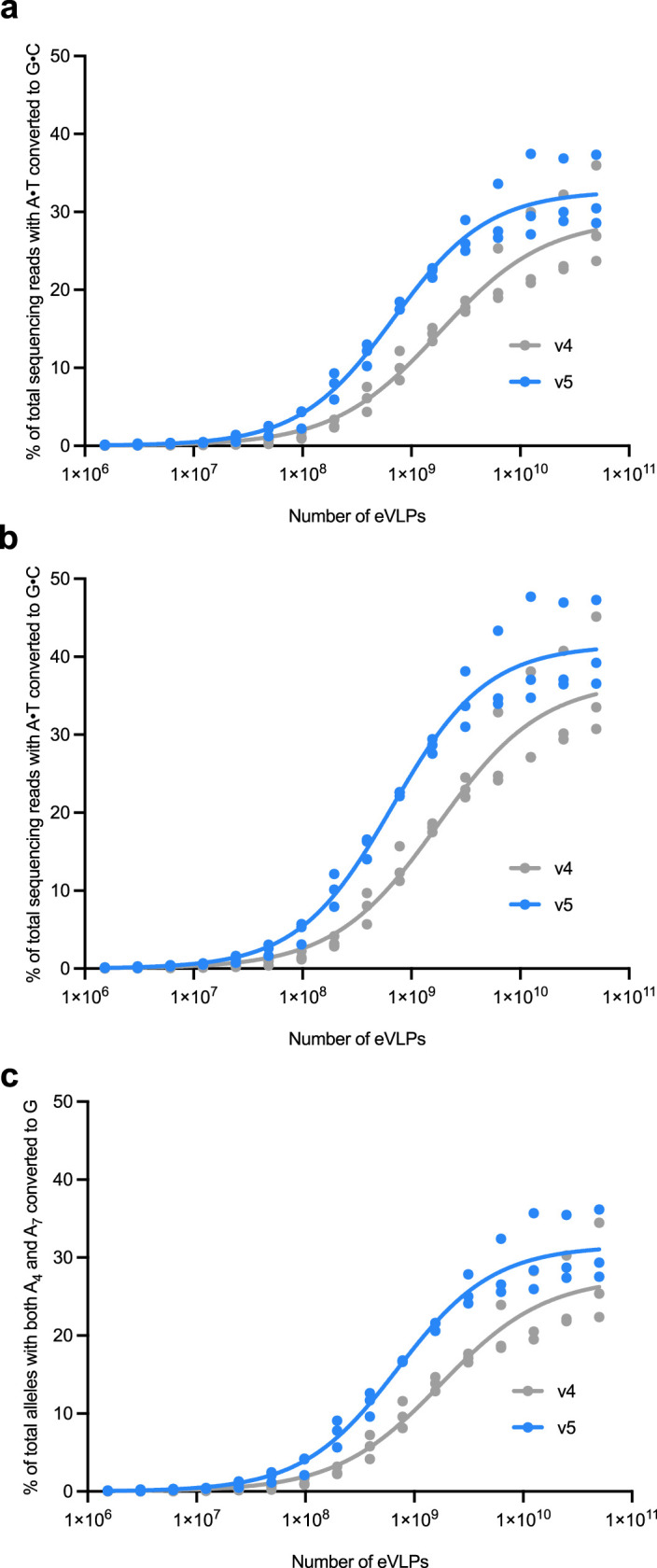
Comparison of v4 and v5 BE-eVLPs in primary human HSPCs across a range of eVLP doses with individual replicate values. **a**, Adenine base editing efficiencies at position A_4_ of the *BCL11A* enhancer site. **b**, Adenine base editing efficiencies at position A_7_ of the *BCL11A* enhancer site. **c**, Adenine base editing efficiencies at both positions A_4_ and A_7_ of the *BCL11A* enhancer site. **a–c**, Dots represent individual replicate values for n = 3 biological replicates. Data were fit to four-parameter logistic curves using nonlinear regression. See also Fig. 4.

To evaluate eVLP-mediated base editing in HSPCs, we transduced healthy human donor HSPCs (Methods) with v4 or v5 BE-eVLPs packaging ABE8e and an sgRNA targeting the *BCL11A* erythroid-specific enhancer locus. We observed substantially higher target adenine base editing efficiencies from v5 BE-eVLPs compared to v4 BE-eVLPs across all doses tested (Fig. 4a–c and Extended Data Fig. 9) Overall, v5 BE-eVLPs exhibited an average 2.6-fold improvement in potency (EC_50_ value) in primary human HSPCs compared to v4 BE-eVLPs (*P* < 0.0001, extra sum-of-squares *F*-test) (Fig. 4b). Notably, the maximum editing efficiency achieved with the highest tested dose of v4 BE-eVLPs was achieved with a 16-fold lower dose of v5 BE-eVLPs (Fig. 4b). These results suggest that v5 eVLPs could greatly simplify the application of eVLPs in large-scale studies by minimizing the dose required to achieve efficient editing, thereby substantially reducing the necessary manufacturing burden. The total number of viable HSPCs detected 48 h after treatment with v4 or v5 eVLPs was comparable to that of untreated HSPC controls, indicating that eVLPs do not substantially perturb HSPC viability (Fig. 4d). Collectively, these results further demonstrate the utility of v5 eVLPs by revealing their improved performance compared to v4 eVLPs in a therapeutically relevant primary human cell type.

### v5 eVLPs exhibit improved cargo packaging and release

**Fig. 5:**
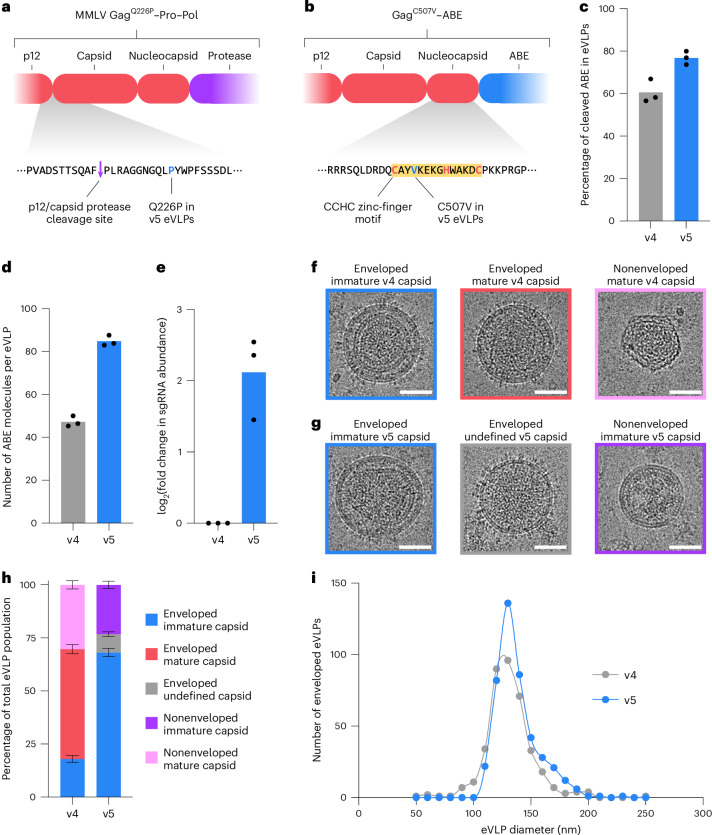
Characterization of v5 eVLPs. **a**, Schematic of the Q226P mutant within the Gag^Q226P^–Pro–Pol in v5 eVLPs, located directly downstream of the internal p12–capsid protease cleavage site**. b**, Schematic of the C507V mutant within the Gag^C507V^–ABE in v5 eVLPs, located within the CCHC zinc-finger motif in the nucleocapsid domain. **c**, Percentage of cleaved ABE cargo detected in v4 or v5 eVLPs. **d**, Quantification of ABE molecules per eVLP by anti-Cas9 and anti-MLV (p30) ELISA (Methods). **e**, Fold change in eVLP-packaged sgRNA abundance measured by RT–qPCR using sgRNA-specific primers, normalized relative to sgRNA abundance in v4 eVLPs. **c**–**e**, Bars reflect the mean of *n* = 3 replicates and dots represent individual replicate values. **f**, Representative cryo-EM images of v4 eVLPs. **g**, Representative cryo-EM images of v5 eVLPs. Scale bars, 50 nm. **h**, Classification and quantification of the types of eVLP capsids observed in the cryo-EM images of v4 or v5 eVLPs. Bars reflect the mean ± s.e.m. of sample proportion for *n* = 551 total v4 eVLPs or *n* = 577 total v5 eVLPs. **i**, Quantification of the particle diameter of every enveloped v4 (*n* = 384) or enveloped v5 eVLP (*n* = 438) observed in the cryo-EM images. Discrete histograms of particle counts were interpolated with an Akira spline curve.

Next, we sought to illuminate the effects of the capsid mutations in v5 eVLPs. The Q226P mutation is located at the N terminus of the capsid domain of Gag, directly upstream of the internal protease cleavage site that separates the capsid and p12 domains following particle maturation (Fig. 5a). Because of its proximity to this protease cleavage site, it is possible that the Q226P mutation alters the rate of cleavage at this site, which could impact capsid formation kinetics to improve packaging large RNP cargos. By contrast, the C507V mutation is located near the C terminus of the nucleocapsid domain of Gag (Fig. 5b). The C507V mutation disrupts the second cysteine in the CCHC zinc-finger motif within the nucleocapsid domain (Fig. 5b) that is known to be required for packaging and replicating viral genomes in wild-type MMLV^65–67^. Because eVLPs lack viral genomes, this CCHC zinc-finger motif is likely no longer required in eVLPs and is instead free to be mutated during selection for improved RNP cargo packaging. The barcoded eVLP evolution system, therefore, identified a capsid mutation that removes a native viral function not used in RNP-delivering eVLPs, further highlighting the benefits of mutating and selecting eVLP capsids to become more optimal for packaging non-native RNP cargos instead of viral genomes.

**Extended Data Fig. 10:**
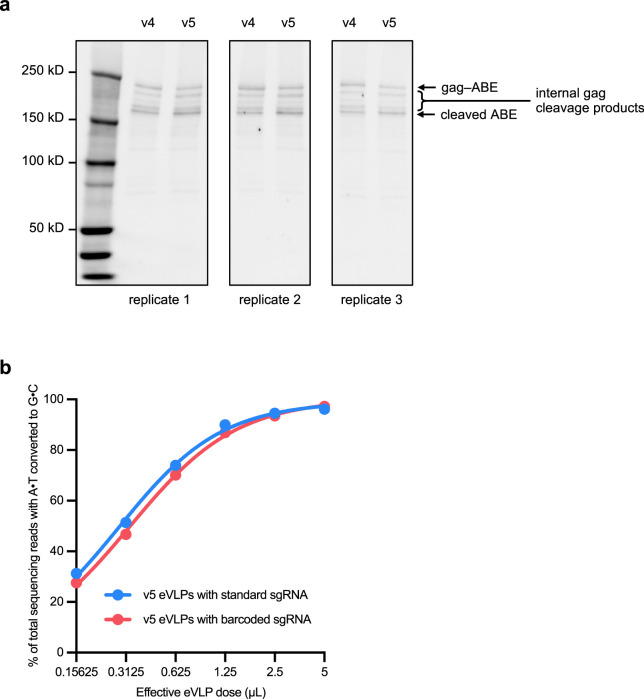
Characterization of v5 eVLPs. **a**, Western blot analysis of lysed v4 and v5 eVLPs using an anti-Cas9 antibody. The full-length non-cleaved gag–ABE fusion is ~247 kD and the cleaved ABE is ~184 kD. Three additional cleavage products, which are generated by protease cleavage at one of three cleavage sites internal to gag (see Extended Data Fig. 2a) and still contain the ABE, were detected at intermediate molecular weights. **b**, Comparison of the potency of v5 eVLPs with barcoded or standard sgRNAs across a range of eVLP doses. Adenine base editing efficiencies at position A_7_ of the *BCL11A* enhancer site in HEK293T cells are shown. eVLPs were produced at a concentration of approximately 5e8 eVLPs/µL. Dots and error bars represent mean ± s.e.m. of n = 3 biological replicates. Data were fit to four-parameter logistic curves using nonlinear regression. Source data

To experimentally characterize the effects of the v5 capsid mutations, we analyzed the protein and sgRNA content of v4 and v5 eVLPs. We previously identified efficient cargo release as a key determinant of eVLP potency^16^. Western blot analysis of lysed eVLPs revealed more efficient cleavage of the capsid–cargo linker in v5 eVLPs compared to v4 eVLPs (Fig. 5c and Extended Data Fig. 10a), indicating that improved cargo release in v5 eVLPs likely contributes to their improved potency. Next, we quantified the number of ABE protein molecules packaged per eVLP by ELISA and observed a 1.8-fold increase in protein packaging in v5 eVLPs compared to v4 eVLPs (Fig. 5d). We also detected a 4.3-fold increase in the sgRNA packaging levels by RT–qPCR in v5 eVLPs compared to v4 eVLPs (Fig. 5e). The combined increases in protein and sgRNA packaging suggest that v5 eVLPs package substantially more active RNPs per particle compared to v4 eVLPs, which likely contributes to their improved potency.

Our previous attempts to improve cargo packaging beyond that of v4 eVLPs resulted in increased protein packaging but not sgRNA packaging^16^. It is, therefore, noteworthy that the v5 capsid mutations evolved to improve RNP packaging and not just protein packaging, likely because barcoded sgRNA abundance was used as the readout for all selections and, thus, the selection system rewarded higher sgRNA packaging levels. We observed that v5 eVLPs are equally compatible with barcoded sgRNAs and standard sgRNAs, consistent with our hope that the activity of evolved eVLPs would not be strongly dependent on the use of barcoded sgRNAs (Extended Data Fig. 10b). Together, these results indicate that v5 eVLPs exhibit improved RNP cargo packaging and release compared to v4 eVLPs, suggesting a mechanistic hypothesis behind their improved potency.

### v5 eVLPs exhibit altered capsid structure and particle sizes

We next sought to further characterize the physical and structural properties of v4 and v5 eVLPs. Previous studies investigated the internal structure of wild-type MMLV particles and observed that wild-type MMLV capsids exist in either an immature or mature state^54,68^. Immature capsids exist before proteolytic processing of Gag and form a single-layered spherical structure inside the viral envelope^54^. Mature capsids are generated after proteolytic processing of Gag and form a multilayered irregular polyhedral structure inside the viral envelope^54^. Mature capsids are generally required for wild-type retrovirus infection and successful nuclear import of the viral RNA genome in infected cells^54,69^. Previous studies have used various high-resolution imaging approaches to readily distinguish the structures and characteristics of mature and immature MMLV capsids^54,68^.

We performed cryo-electron microscopy (cryo-EM) of purified v4 or v5 eVLPs and analyzed the resulting cryo-EM images to classify all observed eVLPs based on their internal capsid structures. Cryo-EM analysis of v4 eVLPs revealed the presence of three distinct structural classes: (1) enveloped immature v4 capsids; (2) enveloped mature v4 capsids; and (3) nonenveloped mature v4 capsids (Fig. 5f). Enveloped immature v4 capsids displayed the characteristic spherical capsid organization found in immature wild-type MMLV capsids (Fig. 5f), including a single thick striated layer that corresponds to the immature conformation of the N-terminal and C-terminal domains of the capsid^54^. By contrast, enveloped mature v4 capsids instead displayed an irregular polyhedral organization and lacked a thick striated layer (Fig. 5f), indicating a transition to the mature conformation of the capsid’s N-terminal and C-terminal domains^54^. Nonenveloped mature v4 capsids displayed a polyhedral shape and are likely not functional through a canonical VLP delivery mechanism because they lack the viral envelope and envelope proteins required for transducing target cells (Fig. 5f). Of all v4 eVLPs detected in the cryo-EM images, 52% contained enveloped mature capsids, 18% contained enveloped immature capsids and 30% contained nonenveloped mature capsids (Fig. 5h).

Cryo-EM analysis of v5 eVLPs revealed the presence of three distinct structural classes that were markedly different from those found in v4 eVLPs: (1) enveloped immature v5 capsids; (2) enveloped undefined v5 capsids; and (3) nonenveloped immature v5 capsids (Fig. 5g). Enveloped immature v5 capsids displayed the characteristic thick striated capsid layer found in immature v4 capsids and immature wild-type MMLV capsids but immature v5 capsids appeared less closely associated with the viral envelope compared to canonical immature capsids (Fig. 5g). Enveloped undefined v5 capsids displayed neither a thick striated immature capsid layer nor an irregular polyhedral mature capsid (Fig. 5g). Nonenveloped immature v5 capsids displayed a spherical shape and are likely not functional through a canonical VLP delivery mechanism because they lack the viral envelope and envelope proteins required for transducing target cells (Fig. 5g). Of all v5 eVLPs detected in the cryo-EM images, not a single v5 capsid with canonically mature morphology was observed (Fig. 5h). Instead, 68% of all v5 particles contained enveloped immature capsids, 8.7% contained enveloped undefined capsids and 23% contained nonenveloped immature capsids (Fig. 5h). These analyses reveal that the capsid mutations in v5 eVLPs substantially alter the capsid structure compared to v4 eVLPs and potentially inhibit capsid maturation.

The absence of mature capsids in v5 eVLPs indicates that, while mature capsids are required for infectious wild-type MMLV particles, mature capsids are not required for eVLP-mediated delivery of BE RNP cargos. Indeed, because v5 eVLPs are more potent than v4 eVLPs, it is possible that immature capsids are not only sufficient for eVLP delivery but are actually more optimal for eVLP delivery than mature capsids. These results are also consistent with a recent report that demonstrated that mature capsids are not required for successful delivery of Cas9 RNP cargos by human immunodeficiency virus-derived VLPs^53^. While the lack of mature v5 capsids suggests a lack of complete proteolytic cleavage at every internal site within Gag^Q226P^–Pro–Pol, we demonstrated above that proteolytic cleavage of the capsid–cargo linker in Gag^C507V^–ABE is still efficient in v5 eVLPs (Fig. 5c and Extended Data Fig. 10a), which ensures efficient RNP cargo release into the transduced cells. These findings provide additional evidence to support the hypothesis that the optimal capsids for RNP-packaging eVLPs are distinct from the canonical capsids in viral RNA-packaging retroviruses.

Lastly, we analyzed the cryo-EM images to characterize the size distribution of enveloped v4 and v5 eVLPs. We observed that v5 eVLPs were overall slightly larger in mean diameter (137 ± 0.87 nm) compared to v4 eVLPs (131 ± 1.06 nm) (Fig. 5i). Furthermore, the size distribution of v5 eVLPs was significantly skewed toward larger particle diameters compared to the size distribution of v4 eVLPs (*P* < 0.0001, Kolmogorov–Smirnov test) (Fig. 5i). The increased size of v5 eVLPs might enable them to accommodate more cargo molecules per particle compared to v4 eVLPs, consistent with our observation above (Fig. 5d). Collectively, these analyses further illuminate the effect of the capsid mutations on various eVLP properties and the differences between v4 and v5 eVLPs that may contribute to the improved potency of v5 eVLPs.

## Discussion

We developed a system for the directed evolution of eVLPs with desired properties and applied this system to mutate and select eVLP capsid mutants with improved properties. This eVLP evolution system, which leverages barcoded sgRNAs to identify eVLP variants that enrich during selections for desired properties, provides a general approach for developing improved eVLPs. By mutating and selecting eVLP capsids toward enhanced eVLP production and transduction, we developed v5 eVLPs that exhibit improved RNP cargo packaging, improved cargo release, distinct capsid structural compositions, increased particle sizes and a 2–4-fold increase in in vitro delivery potency compared to v4 eVLPs.

Because eVLPs consist of multiprotein assemblies in which each component has multiple structural and functional roles, it can be challenging to use rational protein engineering to endow eVLPs with specific properties. Our approach, which used unbiased capsid mutagenesis followed by selections for improved production and transduction, yielded beneficial capsid mutations that would have been very difficult to discover through rational engineering. While VLPs derived from different viruses have been previously described^3,16,26,27,32^, to our knowledge, all previously reported VLPs used wild-type viral capsids and none used capsids that were mutated and selected in the laboratory. Our results suggest that mutated capsids support improved RNP cargo packaging by remodeling native capsid–viral genome interactions and the process of capsid maturation that are likely dispensable in genome-free eVLPs.

The capsid mutation and selection campaign also revealed insights into the properties of eVLPs, illuminating a possible tradeoff between mutations that enhance production versus transduction and the complex interplay between mutations incorporated into the Gag–ABE versus Gag–Pro–Pol constructs. The iterative rediversification and reselection of capsid variants following initial selection would enable the evolution of many synergistic combinations of capsid mutants over multiple generations. In addition to the barcoded library cloning strategy we presented here, which uses oligonucleotide-specified barcode–variant linkages (Extended Data Fig. 2), various other strategies could be used to rediversify eVLP variants in subsequent generations, including gene-wide error-prone mutagenesis^48^ followed by assembly of variants with random barcode sequences and long-read sequencing to decode the assembled barcode–variant linkages. The components of the barcoded eVLP evolution system that we developed and optimized in this work in principle can also be used to implement multigeneration eVLP evolution beyond individual selections and manual combination of surviving mutations described in this study.

In addition to advances in eVLP delivery, our results establish a framework for constructing barcoded eVLP libraries and performing barcoded eVLP selections. The requirement that each producer cell expresses a single combination of barcode and eVLP component variant is critical for maintaining the prescribed barcode–variant linkage during eVLP production. In this study, we achieved this requirement using lentiviral transduction at a low MOI but recombination between lentiviral genomes during virus production might disrupt the barcode–variant linkage^70,71^, decreasing the signal-to-noise ratio in selections. Future implementations of the barcoded eVLP system could use alternative methods such as transposon-mediated integration^72^ or emerging methods for targeted gene integration^73,74^, which might offer benefits relative to lentiviral transduction. Additionally, producing barcoded eVLPs in an arrayed fashion would eliminate any risks of recombination while still enabling useful library sizes and pooled selections.

Our barcoded eVLP evolution system can be used to diversify and select any eVLP component (not just the capsid) for improved performance. In particular, evolving eVLP envelope components could provide more efficient ways to reprogram the cellular tropism of eVLPs. While recent progress has advanced promising strategies for modulating the cell-type specificity of VLPs and related delivery vehicles^16,26,34,52,53,75^, a high-throughput method for evaluating different targeting strategies in eVLPs could reveal insights that are challenging to discover using rational engineering. Barcoded eVLP libraries in principle should be compatible with in vivo selections and, therefore, could be evolved for improved tissue targeting in vivo using approaches similar to barcoded LNP screening^76,77^. Lastly, while we focused on barcoded eVLP evolution, the general strategy we developed for encoding a protein-based delivery vehicle’s identity using packaged barcoded sgRNAs could be applied to evolve other protein-based or peptide-based delivery modalities. Thus, the barcoded eVLP evolution approach described here may be broadly useful for developing delivery vehicles that overcome existing limitations.

## Methods

### Cloning

Plasmids used in this study were cloned using USER cloning as described previously^16^. DNA was amplified by PCR using PhusionU Green Multiplex PCR master mix (Thermo Fisher Scientific; F562L). Mach1 (Thermo Fisher Scientific; C862003) or NEB Stable (New England Biolabs; C3040H) chemically competent *Escherichia coli* was used for plasmid propagation.

### Cell culture

HEK293T cells (American Type Culture Collection (ATCC); CRL-3216) and Gesicle Producer 293T cells (Takara; 632617) were maintained in DMEM + GlutaMAX (Life Technologies; 10569044) supplemented with 10% (v/v) FBS (Gibco). Cells were cultured at 37 °C with 5% carbon dioxide and were confirmed to be negative for *Mycoplasma* by testing with MycoAlert (Lonza; LT07-318).

### eVLP production and purification

eVLPs were produced as described previously^16^. In brief, eVLPs were produced by transient transfection of Gesicle Producer 293T cells. For medium-scale to large-scale preparations, Gesicle cells were seeded in T-75 flasks at a density of 5 × 10^6^ cells per flask. After 20–24 h, cells were transfected using the jetPRIME transfection reagent (Polyplus; 114-75) according to the manufacturer’s protocols. For producing BE-eVLPs or Cas9-eVLPs, a mixture of plasmids expressing VSV-G (400 ng), MMLV Gag–Pro–Pol (3,375 ng), Gag–ABE or Gag–Cas9 (1,125 ng) and an sgRNA (4,400 ng) was cotransfected per T-75 flask. VSV-Gmut-pseudotyped eVLPs were produced as above by replacing the VSV-G-expressing plasmid with pMD2-VSV-Gmut, a gift from M. Birnbaum (Addgene, plasmid 182229). v3 PE2-eVLPs were produced as reported previously^36^ by cotransfection of the following mixture of plasmids: VSV-G (400 ng), MMLV Gag–Pro–Pol (2,813 ng), Gag–MCP–Pol (1,125 ng), Gag–PE (563 ng) and MS2-epegRNA (4,400 ng). v3b PE2-eVLPs were produced as reported previously^36^ by cotransfection of the following mixture of plasmids: VSV-G (400 ng), MMLV Gag–Pro–Pol (2,813 ng), Gag–COM–Pol (2,000 ng), Gag–P3–Pol (422 ng), P4–PE (422 ng) and COM-epegRNA (4,400 ng).

Then, 40–48 h after transfection, producer-cell supernatant was harvested and centrifuged for 5 min at 500*g* to remove cell debris. The clarified eVLP-containing supernatant was filtered through a 0.45-µm PVDF filter. The filtered supernatant was concentrated 100-fold using PEG-it virus precipitation solution (System Biosciences; LV825A-1) according to the manufacturer’s protocols and resuspended in Opti-MEM serum-free medium (Thermo Fisher Scientific; 31985070).

### eVLP transduction and gDNA isolation

Cells were transduced with eVLPs as described previously^16^. Cells were plated for transduction in 48-well plates at a density of 30,000–40,000 cells per well. After 20–24 h, eVLPs were added directly to the culture medium in each well. Then, 48–72 h after transduction, cellular gDNA was isolated as previously reported^16^. In brief, cells were washed once with PBS and lysed in 150 µl of lysis buffer (10 mM Tris-HCl pH 8.0, 0.05% SDS and 25 µg ml^−1^ proteinase K (Thermo Fisher Scientific; EO0492)) at 37 °C for 1 h followed by heat inactivation at 80 °C for 30 min.

### High-throughput sequencing of gDNA

gDNA was isolated as described above. Following gDNA isolation, 1 µl of the isolated DNA (1–10 ng) was used as input for the first of two PCR reactions. Genomic loci were amplified in PCR1 using PhusionU polymerase (Thermo Fisher Scientific; F562L). PCR1 primers for are listed in Supplementary Table 1 under the HTS_fwd and HTS_rev columns. PCR1 was performed as follows: 95 °C for 3 min; 30 cycles of 95 °C for 15 s, 61 °C for 20 s and 72 °C for 30 s; 72 °C for 1 min. PCR1 products were confirmed on a 1% agarose gel. Then, 1 μl of PCR1 was used as an input for PCR2 to install Illumina barcodes. PCR2 was conducted for nine cycles of amplification using Phusion HotStart II polymerase (Thermo Fisher Scientific; F537L). Following PCR2, samples were pooled and gel-purified in a 1% agarose gel using a QIAquick Gel Extraction Kit (Qiagen; 28704). Library concentration was quantified using the Qubit high-sensitivity assay kit (Thermo Fisher Scientific; Q33230). Samples were sequenced on an Illumina MiSeq instrument (paired-end reads; read 1: 200–280 cycles, read 2: 0 cycles) using an Illumina MiSeq 300 v2 Kit (Illumina).

### High-throughput sequencing data analysis for gene editing quantification

Sequencing reads were demultiplexed using the MiSeq Reporter software (version 2.6) (Illumina) and were analyzed using CRISPResso2 version 2.2.14 (ref. ^78^) as previously described^16^. Batch analysis mode (one batch for each unique amplicon and sgRNA combination analyzed) was used in all cases. Reads were filtered by minimum average quality score (*Q* > 30) before analysis. The following quantification window parameters were used: -w 20 -wc -10. Base editing efficiencies are reported as the percentage of sequencing reads containing a given base conversion at a specific position. Prism 10 (GraphPad) was used to generate dot plots and bar plots.

### Lentiviral vector production

HEK293T/17 (ATCC; CRL-11268) cells were maintained in antibiotic-free DMEM supplemented with 10% (v/v) FBS. On day 1, 5 × 10^6^ cells were plated in 10 ml of medium in T-75 flasks. The following day, cells were transfected with 6 µg of VSV-G envelope plasmid, 9 µg of psPAX2 (plasmid encoding viral packaging proteins) and 9 µg of transfer vector plasmid diluted in 1,500 µl of Opti-MEM with 70 µl of FuGENE HD transfection reagent (Promega; E2312). Then, 2 days after transfection, the medium was centrifuged at 500*g* for 5 min to remove cell debris followed by filtration using a 0.45-µm PVDF vacuum filter. The filtered supernatant was concentrated using PEG-it virus precipitation solution (System Biosciences; LV825A-1) according to the manufacturer’s protocols and resuspended in Opti-MEM serum-free medium.

### eVLP-packaged sgRNA extraction

RNA was extracted from eVLPs as described previously^16^. In brief, the QIAmp Viral RNA mini kit (Qiagen; 52904) was used according to the manufacturer’s protocols. Extracted RNA was reverse-transcribed using SuperScript III first-strand synthesis SuperMix (Thermo Fisher Scientific; 18080400) and an sgRNA-specific DNA primer according to the manufacturer’s protocols. The resulting complementary DNA (cDNA) was used as input for standard high-throughput sequencing preparation described above to sequence sgRNA barcodes.

### Barcoded eVLP capsid library vector construction

Oligonucleotide pools containing barcode–capsid variant pairs were synthesized by Twist Biosciences. Oligonucleotide pools were amplified using KAPA HiFi HotStart ReadyMix (Roche Diagnostics; KK2602) supplemented with 3% (v/v) DMSO. Primers for amplification were added to a final concentration of 0.5 µM. Then, 1 ng of oligonucleotide pool template was added per 25-µl reaction. Next, 70–100 ng of the total oligonucleotide pool was minimally amplified to reduce the probability of PCR crossover recombination that could scramble the linkage between barcode sequence and capsid mutant. Oligonucleotide pools were amplified by PCR using the following protocol: 95 °C for 3 min; six cycles of 98 °C for 20 s, 61 °C for 15 s and 72 °C for 1 min; 72 °C for 1 min. Amplified oligonucleotide pools were purified and concentrated using the MinElute reaction cleanup kit (Qiagen; 28104). Concentrated, amplified oligonucleotide pools were assembled with predigested and gel-purified acceptor vector plasmids by Gibson assembly using NEBuilder HiFi DNA assembly master mix (New England Biolabs; E2621L) according to the manufacturer’s protocols. Assembled products were purified using the MinElute reaction cleanup kit (Qiagen; 28104). Electrocompetent cells were generated from NEB Stable (New England Biolabs; C3040H) chemically competent *E.* *coli* by growing single colonies to mid-log phase, collecting cells by centrifugation at 5,000*g* for 1 min at 4 °C, washing with cold 10% (v/v) glycerol and repeating for a total of four washes. Freshly prepared electrocompetent cells were transferred to a chilled 0.1-cm electroporation cuvette (Bio-Rad; 1652089) and mixed with the purified, assembled library plasmids. Cells were electroporated using a time-constant protocol with *t* = 5 ms at 1.5 kV. Electroporated cells were recovered at 37 °C for 25 min with shaking. Recovered cells were plated onto 500-cm^2^ plates containing Luria–Bertani (LB) medium + 1.5% agar supplemented with 100 µg ml^−1^ carbenicillin and incubated for 16 h at 37 °C.

After overnight incubation, colonies were scraped into LB medium and cells were collected by centrifugation. Gibson-assembled library plasmids were purified using a Plasmid Plus maxi kit (Qiagen; 12963) according to the manufacturer’s protocols. The purified plasmids were digested with BsmBI-v2 (New England Biolabs; R0739L) overnight at 55 °C according to the manufacturer’s protocols and the digested product was subsequently purified by performing two successive gel extractions using the QIAquick gel extraction kit (Qiagen; 28704). Purified digests were assembled with the appropriate PCR-amplified inserts using the NEBridge Golden Gate assembly kit BsmBI-v2 (New England Biolabs; E1602L) according to the manufacturer’s protocols. Electroporation, plating and plasmid isolation from transformed colonies were performed as described above. Library quality was assessed using diagnostic digests to confirm uniform plasmid size, Sanger sequencing of 16–32 colonies to verify the correct barcode–mutant linkage and high-throughput sequencing of the barcodes to ensure adequate coverage of all library members. Library cloning was performed separately to generate four distinct sublibraries in which each sublibrary contained every capsid mutant within a 150-bp region. All capsid mutants and corresponding barcode sequences are listed in Supplementary Table 2.

### Barcoded eVLP capsid library selections

Lentiviral libraries were produced as described above. In parallel, Gesicle cells were seeded at a density of 3.4 × 10^6^ cells per T-75 flask. Then, 24 h after seeding, each flask of Gesicle cells was infected with 500 µl of concentrated lentivirus (from 10 ml of viral producer cell supernatant). Next, 24 h after transduction, the medium was changed and puromycin selection was initiated at a final puromycin concentration of 1 µg ml^−1^. Cell viability was monitored and cells were expanded upon reaching confluency. The initial MOI was inferred by counting surviving cells at 1 week after transduction and assuming a doubling time of 24 h. In all cases, MOIs between 0.01 and 0.1 were achieved and an average of 100–300 cells were transduced per library member (Supplementary Table 5).

For production selections, after sufficient expansion of the integrated producer cell library, these cells were seeded for eVLP production in T-75 flasks at a density of 5 × 10^6^ cells per flask. At this time, 5 × 10^5^ cells were collected by centrifugation, lysed in lysis buffer as described above and reserved for sequencing analysis of producer-cell integrated barcode sequences. Then, 24 h after seeding, cells were transfected using the jetPRIME transfection reagent (Polyplus; 114-75) according to the manufacturer’s protocols. A mixture of pUC19 (5,500 ng), VSV-G (400 ng) and MMLV Gag–Pro–Pol (3,375 ng) plasmids was cotransfected per T-75 flask. Then, 40–48 h after transfection, eVLPs were harvested and filtered as described above. The filtered supernatant was concentrated 1,000-fold by ultracentrifugation using a cushion of 20% (w/v) sucrose in PBS. Ultracentrifugation was performed at 26,000 rpm (86,000*g*) for 2 h (4 °C) using an SW28 rotor in an Optima XPN Ultracentrifuge (Beckman Coulter). Following ultracentrifugation, eVLP pellets were resuspended in cold PBS pH 7.4 (Gibco; 10010023). RNA was extracted from purified eVLPs as described above and extracted RNA was reverse-transcribed as described above. The resulting cDNA was amplified by PCR using Phusion HotStart II polymerase using 2 µl of cDNA input per 25-µl reaction and the following conditions: 95 °C for 3 min; 16 cycles of 95 °C for 15 s, 61 °C for 20 s and 72 °C for 30 s; 72 °C for 1 min.

The producer-cell gDNA collected above was purified from crude lysate using an Agencourt DNAdvance kit (Beckman Coulter; V10309) according to the manufacturer’s protocols. The resulting purified gDNA was amplified by PCR using Phusion HotStart II polymerase using 500 ng of gDNA input per 25-µl reaction and the following conditions: 95 °C for 3 min; 30 cycles of 95 °C for 15 s, 61 °C for 20 s and 72 °C for 30 s; 72 °C for 1 min. Illumina barcodes were installed as described above and samples were prepared for sequencing as described above. Samples were sequenced on an Illumina MiSeq instrument (paired-end reads; read 1: 150 cycles, read 2: 0 cycles) using an Illumina MiSeq 150 v3 Kit (Illumina).

For transduction selections, barcoded eVLP capsid libraries were produced and purified as described above. In parallel, HEK293T cells were seeded in 48-well plates at a density of 40,000 cells per well. Then, 18 h after seeding, treated wells were transduced with 20 µl of 1,000-fold concentrated, purified eVLP libraries. Next, 6 h after transduction, cells were trypsinized and washed with PBS. RNA was extracted from cells using the RNeasy Plus mini kit (Qiagen; 74134) according to the manufacturer’s protocols. Extracted RNA was reverse-transcribed and prepared for high-throughput sequencing as described above, with the modification of 23 cycles of PCR1 amplification.

### Barcoded eVLP capsid library selection enrichment analysis

Sequencing reads were demultiplexed using the MiSeq Reporter software (version 2.6) (Illumina). Reads were filtered using fastp^79^ (version 0.20.1) to ensure an average quality *Q* > 30 and using seqkit^80^ (version 2.0.0) to ensure that the reads contained the correct flanking sequences surrounding the 15-bp barcode sequence. The numbers of reads containing each unique barcode sequence were quantified using a custom Python script provided in Supplementary Note 1. For quantification purposes, to account for sequencing errors, any reads that contained a sequence that was within two mismatches of a particular barcode sequence in the library were marked as containing that particular barcode sequence. For calculating barcode frequency enrichments in one population relative to another population (for example, eVLP-packaged sgRNAs versus producer-cell gDNA), raw read counts were converted into reads per million (RPM) with a pseudocount of 1 added for each barcode and fold change values were calculated using the RPM values. Only barcodes that were found in >100 total reads in both preselection and postselection populations were analyzed. Production and transduction enrichment values for each capsid mutant are listed in Supplementary Table 3.

### High-throughput capsid mutant potency assay

To assess the potency of individual capsid mutants independently in a high-throughput fashion, eVLPs were produced in 96-well plates. Gesicle cells were seeded in 96-well plates at a density of 20,000 cells per well. After 24 h, cells were transfected using the jetPRIME transfection reagent (Polyplus; 114-75) according to the manufacturer’s protocols. A mixture of plasmids expressing VSV-G (4.3 ng), evolved or wild-type MMLV Gag–Pro–Pol (36.3 ng), evolved Gag–ABE8e (12 ng) and an sgRNA targeting the *BCL11A* enhancer site (47.3 ng) was cotransfected per well. Edge wells were avoided. Then, 24 h after transfection, HEK293T cells were seeded for transduction in separate 96-well plates at a density of 16,000 cells per well. Next, 48 h after transfecting the Gesicle producer cells, the eVLP-containing supernatant was harvested and pipetted directly onto the seeded HEK293T cells without any additional concentration or purification. A volume of 10 µl of crude eVLP-containing supernatant was used to transduce each well of HEK293T cells. Then, 48 h after transduction, gDNA was extracted in 60 µl of lysis buffer as described above. gDNA was amplified and prepared for sequencing as described above to assess editing efficiency. The fold change in potency relative to v4 eVLPs was calculated by dividing the editing efficiency of the capsid variant by the editing efficiency of v4 eVLPs in the same experiment.

### Culture and eVLP transduction of primary human HSPCs

CD34-enriched granulocyte colony-stimulating factor-mobilized peripheral blood stem cells were obtained from three deidentified healthy adult donors (Fred Hutchinson Cancer Center). Cells were collected under protocol 985.03, which was approved by the human subjects institutional review board at the Fred Hutchinson Cancer Center. All donors provided written consent.

HSPCs were maintained in stem cell medium: X-VIVO-10 medium (Lonza; 04-380Q) supplemented with 100 ng µl^−1^ human stem cell factor (R&D Systems; 255-SC/CF), 100 ng µl^−1^ human thrombopoietin (R&D Systems; 288-TP/CF) and 100 ng µl^−1^ human Flt3 ligand (R&D Systems; 308-FK/CF). HSPCs were thawed using a ThawSTAR CFT2 automated thawing system (Biolife Solutions) according to the manufacturer’s protocol. Immediately after thawing, cells were counted and seeded at a density of 125,000 cells per well in 50 µl of stem cell medium in a round-bottom 96-well plate. Before eVLP transduction, the number of eVLPs per unit volume was determined using the MuLV core antigen ELISA kit (Cell Biolabs; VPK-156) to quantify the number of p30 (capsid) molecules present in each eVLP preparation, assuming a copy number of 1,800 molecules of p30 per eVLP, as previously described^16^. Serial dilutions of ultracentrifuge-purified v4 or v5 BE-eVLPs in PBS were added to each well; each well received 2 µl of eVLPs in PBS. After adding eVLPs, the solution in each well was mixed gently but thoroughly by pipetting. eVLP-transduced HSPCs were immediately incubated at 37 °C for 4 h and then 75 µl of prewarmed stem cell medium was added to each well to achieve a cell density of ~1 × 10^6^ cells per ml.

Next, 48 h after eVLP transduction, the total number of viable cells in a subset of treatment conditions was measured using a NucleoCounter NC-3000 (ChemoMetec) and Solution 13: AO•DAPI staining reagent (ChemoMetec) according to the manufacturer’s protocols using NucleoView NC-3000 software (version 2.1.25.12). On the basis of the measured cell numbers, 80 µl of prewarmed stem cell medium was added to each well to maintain a cell density of 1× 10^6^–2 × 10^6^ cells per ml. Then, 96 h after eVLP transduction, HSPCs were collected by centrifugation at 500*g* for 10 min. Cell pellets were lysed directly for gDNA extraction in 150 µl of lysis buffer as described above and isolated gDNA was sequenced as described above.

### Western blot analysis of eVLP protein content

Western blots to analyze the percentage of cleaved ABE cargo in v4 versus v5 eVLPs were performed as described previously^16^. eVLPs were lysed in Laemmli sample buffer (50 mM Tris-HCl pH 7.0, 2% SDS, 10% (v/v) glycerol and 2 mM dithiothreitol) by heating at 95 °C for 15 min. Protein extracts were separated by electrophoresis at 150 V for 45 min on a NuPAGE 3–8% Tris-acetate gel (Thermo Fisher Scientific; EA03755BOX) in 1× NuPAGE Tris-acetate SDS running buffer (Thermo Fisher Scientific; LA0041). Transfer to a PVDF membrane was performed using an iBlot 2 gel transfer device (Thermo Fisher Scientific) at 20 V for 7 min. The membrane was blocked for 1 h at room temperature with rocking in blocking buffer: 1% BSA in TBST (150 mM NaCl, 0.5% Tween-20 and 50 mM Tris-HCl). After blocking, the membrane was incubated overnight at 4 °C with rocking with mouse anti-Cas9 (Cell Signaling Technology; 14697, 1:1,000 dilution). The membrane was washed three times with 1× TBST for 10 min each time at room temperature and then incubated with goat anti-mouse antibody (LI-COR IRDye 680RD; 926-68070, 1:10,000 dilution in blocking buffer) for 1 h at room temperature with rocking. The membrane was washed as before and imaged using an Odyssey imaging system (LI-COR). The relative amounts of cleaved ABE and full-length Gag–ABE were quantified by densitometry using ImageJ and the percentage of cleaved ABE relative to total (cleaved + full-length) ABE was calculated.

### eVLP protein content quantification

eVLP protein content quantification was performed as described previously^16^. In brief, eVLPs were lysed in Laemmli sample buffer as described above. The concentration of ABE protein in ultracentrifuge-purified v4 or v5 eVLPs was quantified using the FastScan Cas9 (*Streptococcus pyogenes*) ELISA kit (Cell Signaling Technology; 29666C) according to the manufacturer’s protocols. Recombinant Cas9 (*S.* *pyogenes*) nuclease protein (New England Biolabs; M0386) was used to generate the standard curve for quantification. The concentration of MLV p30 protein in purified eVLPs was quantified using the MuLV core antigen ELISA kit (Cell Biolabs; VPK-156) according to the manufacturer’s protocols. The number of ABE protein molecules per eVLP was calculated by determining the ratio of Cas9 molecules to p30 molecules and assuming a copy number of 1,800 molecules of p30 per eVLP as previously described^16^.

### eVLP sgRNA abundance quantification

RNA was extracted from eVLPs and reverse-transcribed as described above. qPCR analysis of the resulting cDNA was performed using a CFX96 Touch real-time PCR detection system (Bio-Rad) with SYBR green dye (Lonza; 50512). The amount of cDNA input was normalized to MLV p30 content and the relative sgRNA abundance per eVLP was calculated as the log_2_(fold change) (Δ*C*_*q*_) relative to v4 eVLPs.

### Cryo-EM sample preparation and imaging

Frozen eVLPs purified by ultracentrifugation were thawed on ice, centrifuged at 14,000*g* for 10 min and resuspended with an equivalent volume of cold PBS. Gentle pipet mixing was performed to ensure complete eVLP resuspension. Grid preparation for 300-mesh R2/1 Quantifoil Cu grids was conducted by glow discharge for 60 s at a set current of 25 mA using a K100X Glow Discharger (EM Sciences). Plunge-freezing was performed using a Vitrobot Mark IV (Thermo Fisher Scientific) set at force 4, 4 °C and 95% humidity; within this instrument, 3 μl of resuspended eVLP sample was drop-cast on a glow-discharged grid and then immediately blotted for 3 or 6 s before plunge-freezing in liquid ethane cooled by liquid nitrogen. Sample grids were then placed in liquid nitrogen for storage.

Cryo-EM was performed using a Talos Arctica G2 (Thermo Fisher Scientific) operating at 200 kV and with a Falcon 3 direct electron detector. Focusing was carried out on carbon film adjacent to a hole and four images at ×92,000 magnification were taken in each hole. Calibrated pixel sizes were 1.605 Å. All datasets were acquired automatically using EPU software (version 2.12.1.2782REL) (Thermo Fisher Scientific).

### Cryo-EM image analysis

Upon visual inspection of cryo-EM images, eVLPs were classified into one of the following types: (1) enveloped immature capsid; (2) enveloped mature capsid; (3) enveloped undefined capsid; (4) nonenveloped immature capsid; or (5) nonenveloped mature capsid. Immature capsids were identified by their paracrystalline lattice shell, indicating the immature conformation of the capsid N-terminal and C-terminal domains^54^. Mature capsids were identified by an irregular polyhedral capsid shape and the lack of paracrystalline shell^54^. Nonenveloped capsids were identified by the absence of an envelope. Classification and sizing were performed manually in ImageJ (version 2.14.0, National Institutes of Health (NIH)). To minimize the potential for selection bias, every discernible eVLP in the set of cryo-EM images was classified and sized.

### Statistical analysis

Data are presented as the mean and s.e.m. No statistical methods were used to predetermine sample size. Statistical analysis was performed using GraphPad Prism software. Sample sizes are described in the figure legends.

### Reporting summary

Further information on research design is available in the Nature Portfolio Reporting Summary linked to this article.

## Online content

Any methods, additional references, Nature Portfolio reporting summaries, source data, extended data, supplementary information, acknowledgements, peer review information; details of author contributions and competing interests; and statements of data and code availability are available at 10.1038/s41587-024-02467-x.

## Supplementary information

Supplementary Information Supplementary Fig. 1 and Note 1

Reporting Summary

Supplementary Tables 1–5 Supplementary Tables 1–5 containing oligonucleotide sequences, protein sequences and raw data.

## Source data

Source Data Extended Data Fig. 10 Uncropped blots.

## Data Availability

The high-throughput sequencing data generated during this study were deposited to the National Center for Biotechnology Information (NCBI) Sequence Read Archive (SRA) database under accession PRJNA1034592. Raw data for individual mutants from the eVLP capsid library selections are provided in Supplementary Table 3. Sequences of eVLP protein components are listed in Supplementary Table 4. Key plasmids from this work were deposited to Addgene for distribution. Other plasmids are available from the corresponding authors on request. Source data are provided with this paper.

## Code availability

The code used for analyzing base editing efficiencies is available from GitHub (https://github.com/pinellolab/CRISPResso2). Custom scripts generated in this study and used for analyzing eVLP selection data are provided in Supplementary Note 1.
