# Supplementary material for: Directed evolution of engineered virus-like particles with improved production and transduction efficiencies
Source: Nat Biotechnol. 2024 Nov 13;43(10):1635–47. doi: 10.1038/s41587-024-02467-x (PMC12085157; doi:10.1038/s41587-024-02467-x)
Supplement: Supplementary file 1 — Supplementary Fig. 1 and Note 1 [file 41587_2024_2467_MOESM1_ESM.pdf]

nature biotechnology

Supplementary information https://doi.org/10.1038/s41587-024-02467-x

Directed evolution of engineered virus-like particles with improved production and transduction efficiencies

In the format provided by the authors and unedited

Supplementary Information

Supplementary Figures.

Supplementary Figure 1. Raw editing efficiency data corresponding to the fold changes in eVLP potency reported in Fig. 3a and Extended Data Fig. 6a.

Supplementary Notes.

Supplementary Note 1. Custom Python script used to analyze barcoded eVLP evolutions.

Supplementary Tables. (provided in a separate file)

Supplementary Table 1. Sequences of primers used for DNA amplification and amplicons analyzed with high-throughput sequencing.

Supplementary Table 2. Capsid mutants and corresponding barcode sequences within the barcoded eVLP capsid library.

Supplementary Table 3. Production and transduction enrichment values for eVLP capsid mutants.

Supplementary Table 4. Full-length eVLP sequences used in this study.

Supplementary Table 5. Total numbers of transduced cells and MOIs for each production selection replicate.

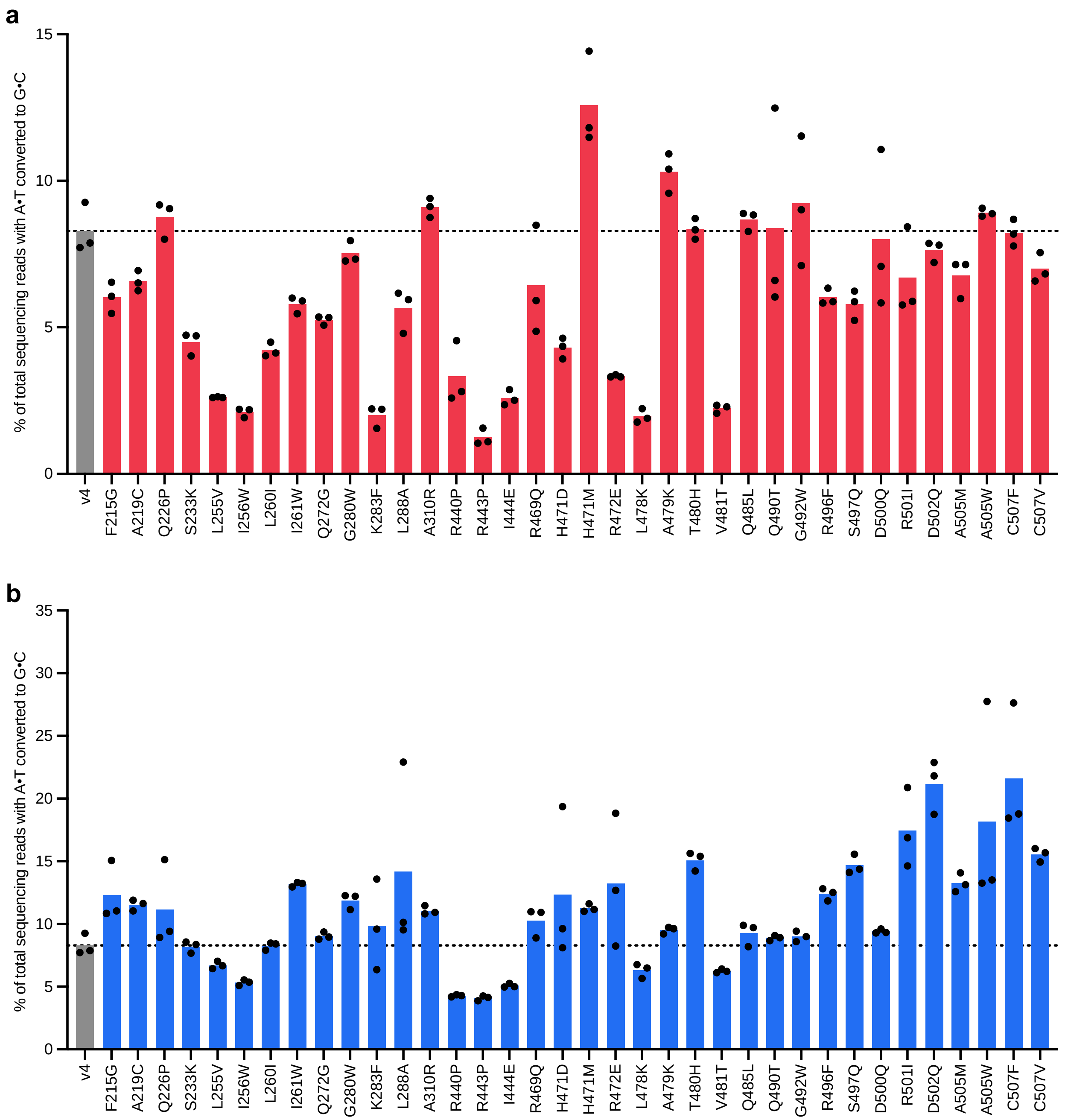

Supplementary Figure 1. Raw editing efficiency data corresponding to the fold changes in eVLP potency reported in Fig. 3a and Extended Data Fig. 6a. a–b, Adenine base editing efficiencies at position A_7 of the *BCL11A* enhancer site in HEK293T cells are shown. Bars reflect the mean of n=3 biological replicates, and dots represent individual replicate values.

Supplementary Note 1. Custom Python script used to analyze barcoded eVLP evolutions.

```
import re
import sys
import pandas as pd

def equal_within_two_mismatches(s1, s2):
    #assume strings are equal length
    pairwise_matches = [1 if s1[i]==s2[i] else 0 for i in range(len(s1))]
    if sum(pairwise_matches) == len(s1) or sum(pairwise_matches) == len(s1)-1 or
sum(pairwise_matches) == len(s1)-2:
        return True
    else:
        return False


#load barcode sequences)
with open(‘barcodes.txt', 'r') as file:
    sequences = file.readlines()
sequences = [sequence.rstrip('\n') for sequence in sequences]

#initialize read counts dictionary
read_counts = {sequence: 0 for sequence in sequences}

#set flanking sequence context based on input (5' context, 3' context)
sequence_context = (str(sys.argv[1]), str(sys.argv[2]))

fastq_filename = str(sys.argv[3])

#open unzipped fastq file, load reads only (every fourth line)
with open(fastq_filename, 'r') as file:
    reads = file.readlines()
reads = reads[1::4]
reads = [read.rstrip('\n') for read in reads]

for read in reads:
    if re.search(sequence_context[0] + 15*'.' + sequence_context[1], read):
        #get library allele sequence
        allele = re.search(sequence_context[0] + 15*'.' + sequence_context[1],
read).group()[len(sequence_context[0]):-len(sequence_context[1])]
        #update counts
        for key in read_counts.keys():
            if equal_within_two_mismatches(allele, key):
                read_counts[key] += 1

#prepare output dataframe
read_counts_df = pd.DataFrame.from_dict(read_counts, orient = 'index', columns =
['counts'])
read_counts_df.sort_values(by = 'counts', ascending = False, inplace = True)
read_counts_df.to_csv(fastq_filename + '_allele_frequency_table.csv')
```
