# Supplementary material for: Directed evolution of engineered virus-like particles with improved production and transduction efficiencies
Source: Nat Biotechnol. 2024 Nov 13;43(10):1635–47. doi: 10.1038/s41587-024-02467-x (PMC12085157; doi:10.1038/s41587-024-02467-x)
Supplement: Supplementary file 2 — Reporting Summary [file 41587_2024_2467_MOESM2_ESM.pdf]

nature portfolio

Corresponding author(s): Aditya Raguram, David R. Liu

Last updated by author(s): Sep 30, 2024

Reporting Summary

Nature Portfolio wishes to improve the reproducibility of the work that we publish. This form provides structure for consistency and transparency in reporting. For further information on Nature Portfolio policies, see our Editorial Policies and the Editorial Policy Checklist.

Statistics

For all statistical analyses, confirm that the following items are present in the figure legend, table legend, main text, or Methods section.

n/a	Confirmed	
☐	☒	The exact sample size (*n*) for each experimental group/condition, given as a discrete number and unit of measurement
☐	☒	A statement on whether measurements were taken from distinct samples or whether the same sample was measured repeatedly
☐	☒	The statistical test(s) used AND whether they are one- or two-sided<br>*Only common tests should be described solely by name; describe more complex techniques in the Methods section.*
☒	☐	A description of all covariates tested
☒	☐	A description of any assumptions or corrections, such as tests of normality and adjustment for multiple comparisons
☐	☒	A full description of the statistical parameters including central tendency (e.g. means) or other basic estimates (e.g. regression coefficient) AND variation (e.g. standard deviation) or associated estimates of uncertainty (e.g. confidence intervals)
☐	☒	For null hypothesis testing, the test statistic (e.g. *F*, *t*, *r*) with confidence intervals, effect sizes, degrees of freedom and *P* value noted<br>*Give P values as exact values whenever suitable.*
☒	☐	For Bayesian analysis, information on the choice of priors and Markov chain Monte Carlo settings
☒	☐	For hierarchical and complex designs, identification of the appropriate level for tests and full reporting of outcomes
☒	☐	Estimates of effect sizes (e.g. Cohen's *d*, Pearson's *r*), indicating how they were calculated

Our web collection on statistics for biologists contains articles on many of the points above.

Software and code

Policy information about availability of computer code

Data collection	MiSeq Reporter software (v2.6) was used on the Illumina MiSeq to demultiplex sequencing data. CryoEM datasets were acquired using EPU software (v2.12.1.2782REL). Cell count and viability data were collected using the ChemoMetec Nucleocounter NC-3000 and the NucleoView NC-3000 software (v2.1.25.12).
Data analysis	CRISPResso2 (v2.2.14) was used to analyze sequencing data for quantifying genome editing efficiency. fastp (v0.20.1) and seqkit (v2.0.0) software packages were used to process library sequencing data, and a custom Python script (provided in Supplementary Note 1) was used to analyze processed sequencing reads for barcode abundances. ImageJ (v2.14.0) was used to perform densitometry of Western blots and analysis of cryoEM images. GraphPad Prism 10 was used for plotting data and statistical analyses.

For manuscripts utilizing custom algorithms or software that are central to the research but not yet described in published literature, software must be made available to editors and reviewers. We strongly encourage code deposition in a community repository (e.g. GitHub). See the Nature Portfolio guidelines for submitting code & software for further information.

Data

Policy information about availability of data

All manuscripts must include a data availability statement. This statement should provide the following information, where applicable:

- Accession codes, unique identifiers, or web links for publicly available datasets
- A description of any restrictions on data availability
- For clinical datasets or third party data, please ensure that the statement adheres to our policy

The high-throughput sequencing data generated during this study are deposited at the NCBI Sequence Read Archive database under PRJNA1034592. Raw data for individual mutants from the eVLP capsid library selections are provided in Supplementary Table 3. Sequences of eVLP protein components are listed in Supplementary Table 4. Uncropped blots from Extended Data Fig. 10 are provided as Source Data. Key plasmids from this work will be deposited to Addgene for distribution. Other plasmids are available from the corresponding authors on request.

Research involving human participants, their data, or biological material

Policy information about studies with human participants or human data. See also policy information about sex, gender (identity/presentation), and sexual orientation and race, ethnicity and racism.

Reporting on sex and gender	HSPCs were obtained from deidentified healthy adult human donors via the Fred Hutchinson Cancer Center.
Reporting on race, ethnicity, or other socially relevant groupings	HSPCs were obtained from deidentified healthy adult human donors via the Fred Hutchinson Cancer Center.
Population characteristics	Deidentified healthy adult human donors
Recruitment	HSPCs were obtained from deidentified healthy adult human donors via the Fred Hutchinson Cancer Center.
Ethics oversight	CD34-enriched G-CSF-mobilized peripheral blood stem cells were obtained from three deidentified healthy adult donors (Fred Hutchinson Cancer Center). Cells were collected under protocol 985.03, which was approved by the human subjects institutional review board (IRB) at the Fred Hutchinson Cancer Center. All donors provided written consent.

Note that full information on the approval of the study protocol must also be provided in the manuscript.

Field-specific reporting

Please select the one below that is the best fit for your research. If you are not sure, read the appropriate sections before making your selection.

☒ Life sciences ☐ Behavioural & social sciences ☐ Ecological, evolutionary & environmental sciences

For a reference copy of the document with all sections, see nature.com/documents/nr-reporting-summary-flat.pdf

Life sciences study design

All studies must disclose on these points even when the disclosure is negative.

Sample size	Sample sizes were n=3 independent biological replicates, which is in accordance with previous literature and standards in the genome editing field (Banskota & Raguram et al. Cell 2022; An et al. Nat. Biotechnol. 2024).
Data exclusions	No data was excluded from the study.
Replication	All experiments were repeated at least once. All attempts at replication were successful.
Randomization	For cell culture experiments, conditions were assigned randomly to wells across 96- or 48-well plates.
Blinding	Sequencing data was analyzed using an automated CRISPResso2 analysis pipeline that does not allow researcher intervention, therefore the researcher was not blinded. Blinding was not necessary for sequencing data collection or other experiments since all samples were processed identically and in parallel (e.g. using multichannel pipettes).

Reporting for specific materials, systems and methods

We require information from authors about some types of materials, experimental systems and methods used in many studies. Here, indicate whether each material, system or method listed is relevant to your study. If you are not sure if a list item applies to your research, read the appropriate section before selecting a response.

Materials & experimental systems

n/a	Involved in the study
☐	☒ Antibodies
☐	☒ Eukaryotic cell lines
☒	☐ Palaeontology and archaeology
☒	☐ Animals and other organisms
☒	☐ Clinical data
☒	☐ Dual use research of concern
☒	☐ Plants

Methods

n/a	Involved in the study
☒	☐ ChIP-seq
☒	☐ Flow cytometry
☒	☐ MRI-based neuroimaging

Antibodies

Antibodies used

Mouse anti-Cas9 antibody (Cell Signaling Technology; 14697)
Goat anti-mouse antibody (LI-COR IRDye 680RD; 926-68070)

Validation

Mouse anti-Cas9 antibody was validated by the manufacturer by Western blot analysis of extracts from 293T cells, mock transfected or transfected with a construct expressing Cas9. Goat anti-mouse antibody was validated by the manufacturer by ELISA and flow cytometry for reactivity with mouse IgG, IgM, and IgA, and by dot blot for minimal cross-reactivity with human, rabbit, goat, rat, and horse serum proteins.

Eukaryotic cell lines

Policy information about cell lines and Sex and Gender in Research

Cell line source(s)

HEK293T, HEK293T/17, and Neuro-2A (N2A) cells were obtained from ATCC. Gesicle 293T cells were obtained from Takara Bio.

Authentication

Commercial cell lines were authenticated by the supplier using STR analysis.

Mycoplasma contamination

All cell lines were tested negative for mycoplasma throughout the duration of this study using the MycoAlert kit (Lonza).

Commonly misidentified lines
(See ICLAC register)

None used.

Plants

Seed stocks

Report on the source of all seed stocks or other plant material used. If applicable, state the seed stock centre and catalogue number. If plant specimens were collected from the field, describe the collection location, date and sampling procedures.

Novel plant genotypes

Describe the methods by which all novel plant genotypes were produced. This includes those generated by transgenic approaches, gene editing, chemical/radiation-based mutagenesis and hybridization. For transgenic lines, describe the transformation method, the number of independent lines analyzed and the generation upon which experiments were performed. For gene-edited lines, describe the editor used, the endogenous sequence targeted for editing, the targeting guide RNA sequence (if applicable) and how the editor was applied.

Authentication

Describe any authentication procedures for each seed stock used or novel genotype generated. Describe any experiments used to assess the effect of a mutation and, where applicable, how potential secondary effects (e.g. second site T-DNA insertions, mosiacism, off-target gene editing) were examined.
