# Supplementary figures and images for: Directed evolution of engineered virus-like particles with improved production and transduction efficiencies
Source: Nat Biotechnol. 2024 Nov 13;43(10):1635–47. doi: 10.1038/s41587-024-02467-x (PMC12085157; doi:10.1038/s41587-024-02467-x)

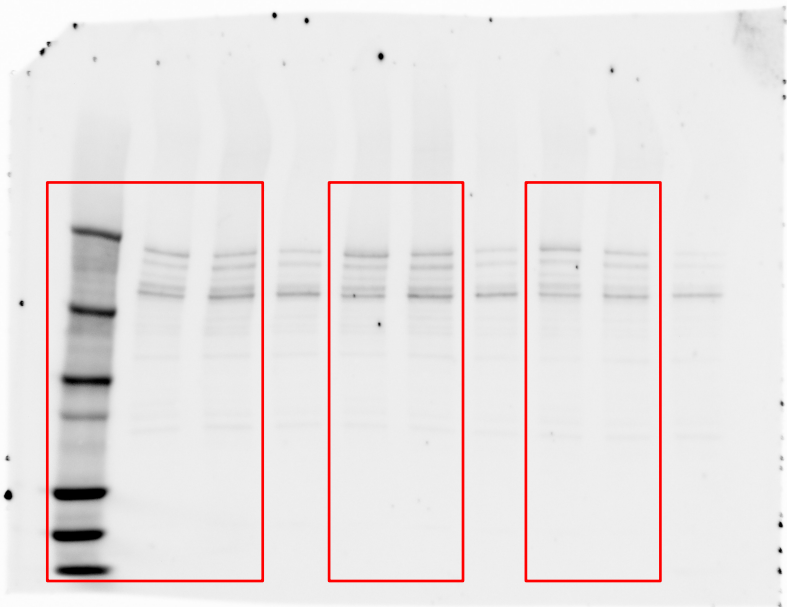

Supplement: Supplementary file 4 — Uncropped blots. [file 41587_2024_2467_MOESM4_ESM.pdf]
